# Greener active pharmaceutical ingredients: perspectives of European healthcare professionals

**DOI:** 10.1186/s12913-026-15145-2

**Published:** 2026-09-04

**Authors:** Neele Puhlmann, Yannick Heni, Rodrigo Vidaurre, Caroline T. A. Moermond, Klaus Kümmerer, Tiina M. Sikanen, Oliver Olsson

**Affiliations:** 1 Institute of Sustainable Chemistry, Leuphana University of Lüneburg, Universitätsallee 1, 21335 Lüneburg, Germany; 2 Ecologic Institute, Pfalzburger Strasse 43/44, 10717 Berlin, Germany; 3 Centre for Safety of Substances and Products (VSP), National Institute for Public Health and the Environment (RIVM), P.O. Box 1, Bilthoven, 3720 BA The Netherlands; 4 Department of Environmental Science, Radboud University Nijmegen, Nijmegen, The Netherlands; 5 Faculty of Pharmacy, University of Helsinki, Viikinkaari 5E, Helsinki, 00790 Finland

**Keywords:** Ecotoxicity, Environment, Impact, Interview, Pharmaceutical, Risk, Stakeholder

## Abstract

Pharmaceutical residues resulting from patient excretion, incorrect disposal and manufacturing of medicines contribute to environmental pollution and related risks. Designing active pharmaceutical ingredients of inherently lower environmental risk (‘greener APIs’) is one potential solution to this issue. To assess the current level of preparedness to adopt potentially greener APIs as a part of more sustainable medical care, the perspectives of healthcare professionals on greener APIs were evaluated by investigating related opportunities, barriers and the needs to overcome these barriers. Semi-structured interviews were conducted with professionals working in the healthcare sector in European countries between February and April 2024 (n = 16). The interviewees included doctors (3), pharmacists (3), experts in procurement (3), reimbursement (1), market authorisation (2), healthcare provision (2) and industry’s sustainability reporting (1), as well as a professional working for an environmental NGO (1). Data was evaluated using qualitative content analysis. Interviewees’ answers showed that some strategies already exist to account for environmental aspects in decision-making, e.g. by adding environmental criteria to the tenders of procurement agencies. Criteria on environmental properties of APIs can be expected to play a more significant role in the decision-making of HC professionals in future. Balancing molecular properties related to patient benefit and risk against environmental properties could be product specific. Frequently expressed needs to enable this include: access to reliable scientific data and evidence of environmental impacts of APIs, legislative and regulatory frameworks and guidance to ensure environmental properties are assessed and weighed in a centralised and harmonised manner. The evident interest among HC professionals to consider environmental properties in their decision-making may stimulate data sharing by pharmaceutical companies in the short-term and efforts to design greener APIs in the long-term. The marketing authorisation process is viewed as the main intervention point in the life cycle of pharmaceuticals to strengthen considerations of environmental risks. The authors conclude that future research should target the identified barriers and their solutions.

**Supplementary Information:**

The online version contains supplementary material available at https://doi.org/10.1186/s12913-026-15145-2.

## Highlights

The study evaluates on the preparedness to adopt greener APIs into use.

Qualitative interview study with 16 European healthcare (HC) professionals.

Findings show interest by HC professionals to minimise environmental impacts.

Barriers and needs to consider environmental criteria were identified.

**Supplementary Information:**

The online version contains supplementary material available at https://doi.org/10.1186/s12913-026-15145-2.

## Introduction

Pharmaceutical residues, including active pharmaceutical ingredients (APIs), their human metabolites and environmental transformation products (TPs), are detected in the aquatic environment globally, and mostly associated to patient excretion [7, 14, 20, 21, 58]. In many regions, including the European Union (EU), current wastewater technologies are not fully effective in removing pharmaceutical residues from municipal wastewaters, if in place at all.

Environmental pollution has been linked to long-term adverse impacts on ecosystem functions and biodiversity [9]. The planetary boundary of the safe operating space for novel entities^1^ introduced into the environment (including environmental pollutants such as active pharmaceutical ingredients) has been exceeded by humanity. Annual production and environmental releases of such novel entities are increasing too rapidly with respect to the global capacity of assessment and monitoring [36, 46, 49].

In the EU, pharmaceuticals have been added to the priority substances list of the Water Framework Directive (2000/60/EC) and the environmental risk assessment (ERA) of pharmaceuticals within their authorisation procedure has been strengthened in the new Pharmaceutical legislation. These legislative frameworks focus on identifying and managing risks once a compound has been selected for development or is on the market. Designing and using APIs that are safe and efficacious for patients and at the same time pose a low environmental risk, e.g., because of better environmental biodegradability (‚greener APIs’, [24, 27]) may help to reduce the risks related to Pharmaceuticals in the Environment (PiE) (Chemical Strategy for Sustainability [12], EU Strategic Approach to PiE [11]). So far, the environmental sustainability programs of pharmaceutical industries have mostly focussed on improving waste management or reducing the environmental impacts of their own operations [44]. Recently performed interviews with R&D experts of pharmaceutical companies have however shown that they consider it possible to integrate environmental parameters into established screening cascades of the drug discovery process if certain requirements are met, such as the availability of suitable test methods [41]. The integration of further parameters would enable research teams to optimise environmental and pharmacokinetic-pharmacodynamic characteristics in parallel and, ideally, find a balance between the contributing parameters. Building on that, Vidaurre et al. [56] investigated the potential opportunities and challenges of an API design approach that simultaneously optimises drug efficacy and safety as well as environmental impact potential. It was stressed that nuanced approaches are needed to achieve this.

The ultimate adoption of such greener APIs in healthcare (HC) further depends on numerous decision-making processes in the entire HC sector. The Dutch chain approach, for example, shows that many stakeholders need to collaborate on solutions to avoid paralysis by the complexity of this issue [29]. It is necessary to incorporate the knowledge, experiences, needs and concerns of all relevant stakeholders into the adaptation of greener APIs along the whole life cycle of pharmaceutical products. Greener APIs have to be designed. They also need to be marketed, procured, reimbursed, prescribed and used. The relevant stakeholders in the context, as identified by Heni et al. [19], include health sector organisations, pharmacists, doctors and patient organisations, companies working in the pharmaceutical sector, authorities and regulators, universities and research organisations, NGOs and the water sector.

The aim of this study was to assess the current level of preparedness to include greener APIs as a part of more sustainable medical care, based on the perspectives of the afore-mentioned HC professionals, and to identify any opportunities, barriers as well as needs that should be addressed. Semi-structured interviews were conducted among professionals working in HC in European countries (*n* = 16), including doctors, pharmacists, experts in procurement, reimbursement, market authorisation, healthcare provision and industry’s sustainability reporting, as well as a professional working for an environmental NGO. Data was analysed using qualitative content analysis. While the findings of interview studies should be contextualised within the scope of the sample, they can contribute meaningfully to understanding a complex topic [57].

## Methods

The views and attitudes of HC professionals were collected via interviews, since in this manner, their expertise and experiences can offer valuable insights that are not necessarily captured by large and generalised surveys. Interviewees were asked to answer the questions as representatives of their organisations. A combination of structured and semi-structured interview methods was applied, because in this manner, perceptions and attitudes on complex and scarcely studied topics can be better evaluated [48]. Therefore, the questionnaire included also open-ended and optional follow-up questions (supplementary information, SI).

### The questionnaire for the interviews

The questionnaire (Table 1) aimed to investigate perspectives of stakeholders of the healthcare sector on the topic of greener APIs, so as to understand the barriers and opportunities to incorporate this aspect (e.g. favouring greener APIs in the healthcare sector) in their work.

The study’s research question centred on the feasibility of the uptake of greener APIs and understanding the potential role of healthcare organisations in such an uptake. The analytical point of entry was the healthcare organisations’ decision-making processes related to pharmaceutical selection or use. The questionnaire’s first section aimed to understand the position and role of the organisation in the pharmaceutical lifecycle and its current decision-making processes related to pharmaceutical selection or use. The second section focused on the current and the potential future role of environmental criteria in this decision-making, with a particular focus on criteria related to environmental impacts after use by patients. The third section addressed factors that influence the feasibility of introducing environmental criteria in decision-making, such as the role of existing sustainability guidelines and policies, and it addressed possible requirements for an uptake of environmental criteria. In addition, the interviews aimed to assess the current level of awareness of the topic among interviewees and their preparedness to include greener APIs as a part of more sustainable medical care along the lifecycle of medicines. This includes related opportunities, barriers and needs (to overcome these barriers).

Development of the interview guide was based on the research question and the analytical point of entry (decision-making processes that the interviewees were involved in). Questions were developed based on expert experience of the study authors.

The developed questionnaire was reviewed by members of the PREMIER project (a public-private partnership project working on ‘Prioritisation and Risk Evaluation of Medicines In the EnviRonment’) [39] to validate the scope and the level of details in the questions and to ensure good comprehensibility. Three review rounds were held with PREMIER partners (participants in Work Package 4.2). Draft versions of the questionnaire were shared with partners one week before virtual meetings in which feedback was discussed and consolidated in consensus decision-making. The feedback was then incorporated into an updated version of the questionnaire, then shared and discussed again. A total of ten reviewers (one pharmacist, nine environmental toxicologists) from eight organisations (one university, one public research centre, one national environmental authority, five pharmaceutical industry partners of PREMIER) participated in the review process. Revisions led to simplification of questions and improving the formulation of the questions on trade-offs with other criteria. No piloting of the interview questionnaire was conducted. Table 1 presents and overview of the three sections outlined above, the aim(s) of each section, and lists the corresponding questions.

**Table 1:** Overview of questionnaire sections, their aims, and interview questions

Questionnaire section and aim(s)	Interview questions
**Section 1**: Setting the scene and understanding the decision-making processes of the interviewees’ organisations in general (10 min) **Aim**: To understand interviewees’ roles and related activities (a prerequisite for Sections 2 and 3)	Q1: Please describe your organisation regarding: a) its position in the life-cycle of pharmaceuticals b) how its mandate is related to the selection/supply/use of pharmaceuticals.
	Q2:Please describe your organisation’s decision-making relating to the selection/use of pharmaceuticals. (E.g.: How do you decide which pharmaceutical is selected/used?)
**Section 2**: The current and future role of environmental considerations in this decision-making (35 min) **Aim**: To study the perceptions and attitudes on environmental criteria, specifically on criteria for post-use environmental impacts	Q3:Do environmental aspects related to pharmaceuticals play a role in your decision-making regarding selection/use of pharmaceuticals? If “yes”: a) Which ones, and which rationale was behind the uptake of these aspects? b) How are these environmental aspects weighed in comparison to other criteria considered in the selection of pharmaceuticals?
	Now specifically on pharmaceuticals in the environment (after use/excretion) and effects:
	Q4:What is your organisation’s knowledge regarding the topic “Pharmaceuticals in the environment and their effects” and potential measures?
	Q5:Do post-use environmental impacts of pharmaceuticals play a role in your organisation’s decision-making? a) If “yes”: Which impacts (most important) do you consider? b) If “no”: Are considerations specific to such impacts expected to be included in your organisation’s future decision-making? • If “yes”: Which impacts may become relevant? Q5a: (Only if your response to question Q5 was “yes” and your organisation is familiar with the aspects listed below.) Which parameters related to post-use environmental impacts may become relevant in your decision-making? Examples could be: • Environmental Risk: PEC/PNEC • Environmental exposure: emissions (e.g. concentrations in effluent at production site), environmental concentrations • Hazardous properties: PBT, vPvB /PMT, vPvM • Substance classification according to regulation, such as CLP and/or REACH • Other parameters a) Which drivers do you see behind your answer?
	Q6:How important would you consider parameters on post-use environmental impacts when compared to other criteria, e.g., costs, dosing frequency, patient safety? a)Would you follow the same decision-making logic when (a) a pharmaceutical has been associated to a confirmed environmental impact due to post-use releases and when (b) a pharmaceutical (despite a hazard classification) is concluded to represent no risk to the environment (e.g., environmental exposure remains lower than the level that could induce an impact)?
	Q7:Do you see risks for undesired consequences when selecting one API over another? a) If “yes”: Please describe the kind of risks. b) What would be required to avoid such risks?
**Section 3**: Factors that determine the feasibility of introducing environmental considerations in decision-making (15 min) **Aims**: (1) To identify the needs of HC professionals to consider environmental criteria, (2) To identify the role of the different stakeholder groups in promoting the adaptation of greener APIs.	Q8:Do your sustainability guidelines or policies refer to the use of pharmaceuticals? Is there any concept/approach in your organisation towards this, e.g. by selecting/prioritizing greener APIs?
	Q9:What would be needed for your organisation to consider environmental aspects of APIs in future?
	Q10:What kind of expertise is needed for assessing and weighing the various types of criteria, e.g. criteria for pharmaceutical’s application such as efficacy and safety, and criteria related to post-use environmental impacts? a) Assuming expertise to perform a holistic assessment is available: Which healthcare actor(s) should perform the assessment (partly or fully) of post-use environmental impacts and/or the weighing of all different criteria? • Doctors • Pharmacists • Hospitals • Pharmaceutical Industry • Health system procurement agencies • Health insurance companies • Authorities responsible for marketing authorisations • Authorities involved in healthcare provision • Environmental authorities • Others

Additional details (life-cycle figure for Q1, figure with overview of sustainability dimensions and impact categories for Q3, examples for Q6) are included in the complete interview questionnaire in the SI

### Interview request and interviewees

The interview request was sent via email to 41 HC professionals in total, including representatives working on international (3), Europe-wide (10), or national tasks (28), and covering nine different countries in North-western Europe (NO, FI, SE, DK, DE, NL, BE, UK).

The aim was to cover a broad spectrum of stakeholders, including:

- doctors,
- pharmacists,
- health system procurement agencies,
- health insurance companies,
- authorities responsible for marketing authorisation (MA),
- authorities involved in HC provision,
- society representatives / patient organisations,
- environmental NGOs, and.
- professionals from industry responsible for sustainability reporting.

The selection of these stakeholder groups was based on the previous work by Heni et al. [19], aiming to have representatives along the life cycle of pharmaceuticals, with emphasis on the distribution and use phases. Initially, the target was to interview at least 2–3 representatives of each group (*n* = 18–27 in total). It was however not possible to include interviewees from all EU countries under every role in the life cycle. We therefore focussed on finding interviewees from countries that are already actively addressing some aspect(s) of risk mitigation for pharmaceuticals in the environment, so they’d have a basic knowledge of the topic.

Study authors, supported by PREMIER project partners, mapped relevant organisations against stakeholder groups. Relevant EU-level organisations, comparatively few in number, were identified first; contact details were identified where possible using personal contact information in literature or thanks to project partners’ contacts. National-level organisations and relevant people within them were identified in a similar process, with more reliance on partners’ networks in this step. Some interview partners were identified, when the first contacted person declined but recommended contacting e.g. a colleague. The invitees were selected from this larger list of contacts (purposive sampling method, [48]), where an equal representation among different stakeholder groups was aimed for. The interview request was accompanied by a short background summary on the topic ‘Pharmaceuticals in the Environment’ and the PREMIER project, including the introduction of the interview study (see SI). If there was no response to the interview request, up to two reminders were sent within 2 weeks. Combining the method of purposive sampling with theoretical sampling [15, 45], interviews were held with professionals in several rounds to enable an evolving interviewing process and the build-in of additional heterogeneity into the sample (i.e., the group of interviewees). Additional rounds of interview contacts were necessary as interview requests were not always answered and sometimes were declined. For the first round of interviews, professionals involved with associations and active in different European countries, or even on the European level, were prioritised. This was to ensure that the results would not be polarised to only one type of HC delivery system, such as Bismarck or Beveridge types in Germany or Sweden, respectively [54].

### Conducting the interviews

The interviews (*n* = 16) were conducted online between February and April 2024 using the video conference platform Zoom, to enable cost efficiency and enhanced long-distance participation [18, 53]. The interviewer team included one person from Leuphana University (section ii) and one alternating person from the three-membered team from Ecologic Institute (sections i and iii). This two-to-one interview method was applied to create a lively atmosphere and to have a powerful effect on rapport [31]. While one interviewer was leading the interview, the other interviewer ensured that the questionnaire’s structure was followed. The interviews lasted on average 66 min (ranging between 40 and 128 min). In three cases out of 16, two interviewees of the same organisation were present. Otherwise, the interviews were performed with one interviewee at a time.

Before starting the interview, the interviewees received the privacy policy and agreed to the recording for the purpose of generating an automatic transcript using a transcription software.

The overall aim was to achieve data saturation in all topics (i.e., the degree to which new data repeat what was mentioned in the previous data). However, due to the heterogeneous background and responsibilities of the HC professionals interviewed as well as the country specific differences in the HC systems, saturation was not achieved with respect to all topics. Nevertheless, the interview results were considered valid to enable drawing initial conclusions about the perspectives of HC professionals on the environmental considerations and their significance for the field. To respond to the limited number of interviews, certain questions were explicitly asked in such a way that the interviewee was asked to not only describe their own personal position, but also that of their professional group.

### Qualitative analysis using MAXQDA 2022

The recorded interviews were transcribed word for word using the transcript software packages Otter.ai (for English) and Trint (for German). The automatic transcripts were checked (proofread) and pseudonymised before further qualitative analysis.

The software MAXQDA 2022 was used to perform a directed content analysis by using codes in the transcripts that were defined before and during data analysis [22]. A code was applied to reflect the answers to a specific question or sub-question (code scheme in SI). Codes thus implemented in detail the questionnaire’s structure, allowing to assign relevant statements in other parts of the interview to the relevant questionnaire’s sub-questions. The unit of coding was the sentence and the paragraph level. Statements established to be out of scope of the study (code “out of scope”) were identified as such by comparing them with the (sub-)question’s theme and objective. A review was performed of out-of-scope statements as identified in the first step of the analysis, additionally checking for their relevance before deciding whether this information was relevant for the research questions or not (code “not relevant”). These typically included statements related to interview admin and process.

The results related to specific questions in Section 2 (Q3 and Q5, see Table 1) and Section 3 (questions Q8-Q10, see Table 1) were further analysed through an iterative, data-driven coding process. Whenever a new coding emerged, earlier responses were revisited to ensure consistency. Next, inductive content analysis was applied to the empirical data generated [52]. For each question, related codes were clustered into broader themes, allowing the themes to develop based on interviewees’ responses, and the data was summarised accordingly. The generation of transcripts and the inductive content analysis were performed by one researcher (NP) to ensure consistency. The coding and the results of the initial analysis were discussed and written together with two interviewers from Ecologic (YH, RV) to ensure objectivity. The draft coding of interviews by NP was reviewed by YH and RV and agreed on by these three authors, ensuring consistent use of codes.

The results of the systematic evaluation are presented anonymised and generalised in the next section, so that statements cannot be traced back to any individual or organisation.

## Results from interviews with HC professionals

Interview requests were accepted by 16 professionals, i.e., 39% of the invited group (Table 2), covering all contacted stakeholder groups except for representatives of the societal sector / patient organisations (eight invited, none accepted). All 16 professionals who accepted the invitation were from the networks of the PREMIER members and a further contact person. As can be observed from Table 2, the pool was geographically biased toward North-Western Europe.

**Table 2:** List of interviews, assigned to a stakeholder group and specified by the role of the interviewee

Interviewed stakeholder group (Country in alphabetical order) Nr. invitation sent (accepted / not accepted)		Stakeholder code	Role of interviewee
1	Doctors (BE, DE, IT) Total invited 5 (accepted 3 / not accepted 2)	Doc-1	Chairman of a regional medical association
		Doc-2	Professor in pharmacology of a medical school, university hospital
		Doc-3	Scientific association doing educational and informational activities for physicians
2	Pharmacists (Europe, FI, SE) Total invited 4 (accepted 3 / not accepted 1)	Pharm-1	Pharmacist
		Pharm-2	Pharmacy association
		Pharm-3	Pharmacy association
3	Health system procurement agencies (DK, FI, NO) Total invited 4 (accepted 3 / not accepted 1)	Proc-1	Senior medical officer, university hospital
		Proc-2	Responsible for procurement of hospitals at national level
		Proc-3	Sustainability specialists for procurement
4	Health insurance companies (DE) Total invited 5 (accepted 1 / not accepted 4)	HI-1	Health insurance company, reimbursement
5	Authorities responsible for marketing authorisation (FI, SE) Total invited 2 (accepted 2 / not accepted 0)	MA-1	Medicines agency, quality assessment
		MA-2	Medicines agency, ERA and advisor/communication
6	Authorities involved in HC provision (SE, UK) Total invited 8 (accepted 2 / not accepted 6)	Prov-1	Drug and Therapeutics Committee (DTC) network, former chairman of a DTC (region)
		Prov-2	Consultant in pharmaceutical public health, environmental protection activities beyond
7	Society representatives / patient organisation Total invited 8 (accepted 0 / not accepted 8)	-	-
8	Environmental NGO (Not mentioned for privacy policy reasons) Total invited 4 (accepted 1 / not accepted 3)	NGO-1	Think tank, acting on regulatory level concerning entire lifecycle
9	Industry: responsible for sustainability reporting (country not mentioned for private policy reasons) Total invited 1 (accepted 1 / not accepted 0)	Ind-1	Senior manager sustainability reporting, accompanied by environmental risk assessor

Up to three professionals per stakeholder group from different countries were interviewed. The stakeholder code is used in the following as abbreviation for the interviewed professional

In the following, the interview results are reported separately for each of the three sections of the interview questionnaire.

### Section 1 - Role and activities of the interviewed professionals in the lifecycle of medicines

**Fig. 1:**
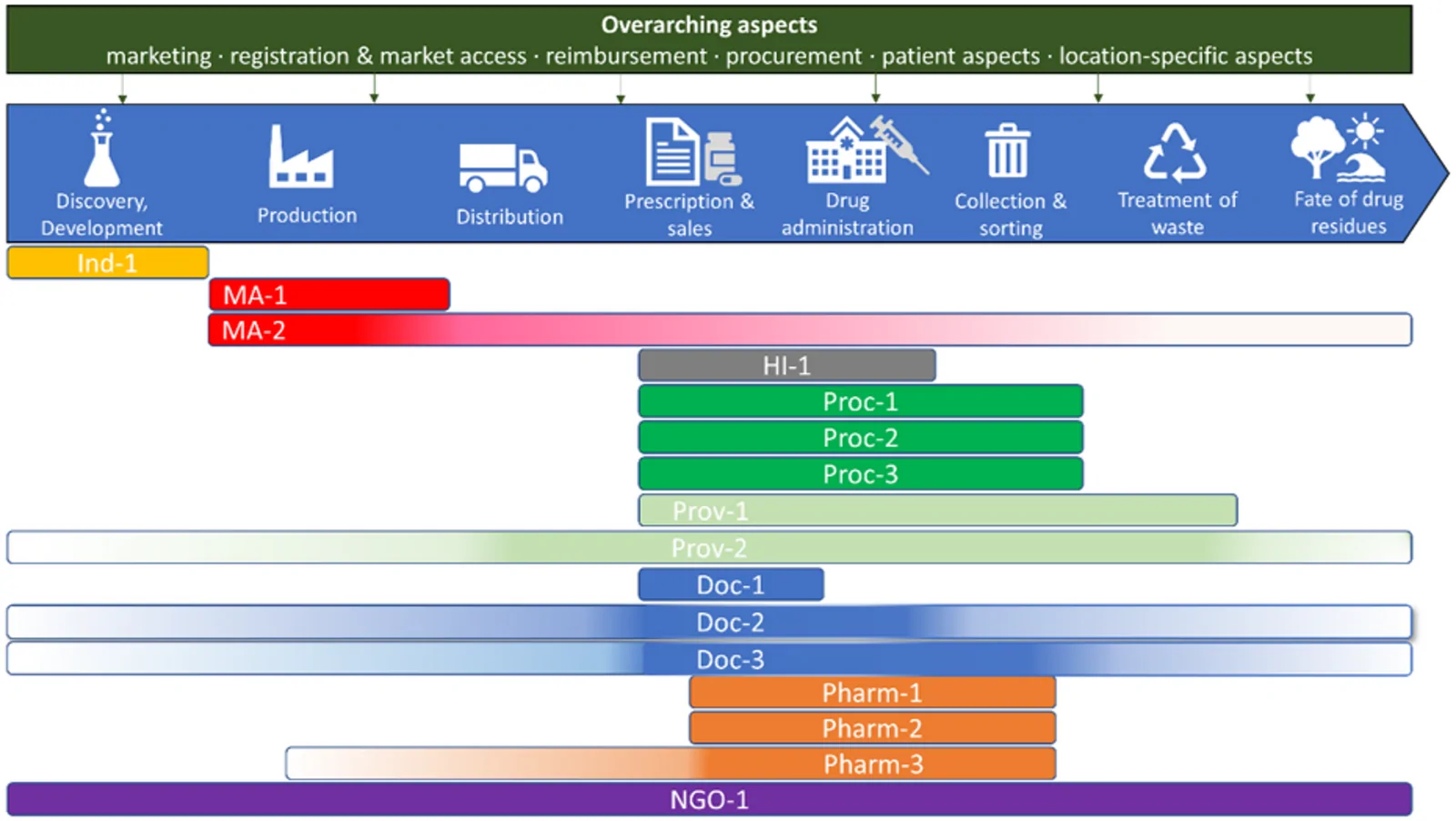
Position in lifecycle of pharmaceuticals and the extent of professionals’ activities. Each bar represents interviewee’s position and the range of activities (according to the interviewees themselves). Bars with colour shading visualise that there are secondary activities (lighter area) next to the core area of interviewee’s position (more saturated area). Upper part of the figure is inspired by van Wilder et al. [55]. The abbreviations (Ind, MA, HI, etc.) are introduced in Table 1

To evaluate the study design, the interviewees were first asked for their roles and activities in relation to the lifecycle of medicines (Questions 1 and 2, see Table 1). As illustrated in Fig. 1, the interviewees’ main responsibilities resonated well with the aimed stakeholder group (i.e., the distribution and use phases of the lifecycle). Some interviewees (5/16) mentioned a core area and secondary activities beyond the distribution and use phases (Fig. 1, shading colour in the bars). For example, interviewed doctors are primarily active from ‘prescription & sales’ to ‘collecting & sorting”, but secondary activities can also relate to other lifecycle stages, e.g., up to the ‘fate of medicines’ residues’ due to collaborative work in a hospital setting.

### Section 2 - Significance of environmental considerations in the decision-making process currently and in future

#### Perceived knowledge in interviewees’ professional group on *PiE*,* related effects and potential measures*

To evaluate the perceived level of knowledge, interviewees’ were asked to self-evaluate the awareness on the topic ‘*PiE*,* related effects and potential measures*’ within their professional group or team (Question 4; see Table 1). The level of interviewees’ perceived knowledge varied from no/little awareness to very high, reflecting the variations between the interviewed stakeholder groups. According to the doctors (3/3), their colleagues are not at all or barely aware of this topic. However, according to an interviewee involved in the basic education in medicine, awareness is increasing as the result of newly developed teaching materials. This is also applicable to pharmacists (3/3), who mentioned that there is (increasing) awareness due to the implementation of environmental aspects in educational programs. The interviewees working at procurement agencies (3/3), a health insurance company (1/1), an environmental NGO (1/1), and authorities involved in HC provision (2/2) stated that awareness about this topic exists in their organisations, but knowledge is limited and only available through third parties. The interviewees working at a pharmaceutical company (1/1) or authorities responsible for market authorisation (2/2) estimated that the level of knowledge is good or very good at their organisations. It should be noted however that these are the perceptions of a limited number of individuals and the findings should not be generalised. In a pharmaceutical company, for example, the level of knowledge can differ between departments that do not necessarily collaborate.

#### Current role and future perceptions of environmental aspects, including post-use environmental impacts

Interviewees were asked about their views on the importance of environmental aspects in general, that of post-use environmental impacts in particular, their views on related priority areas as well as future perceptions (Questions 3, 5 and 5b, respectively).

**Fig. 2:**
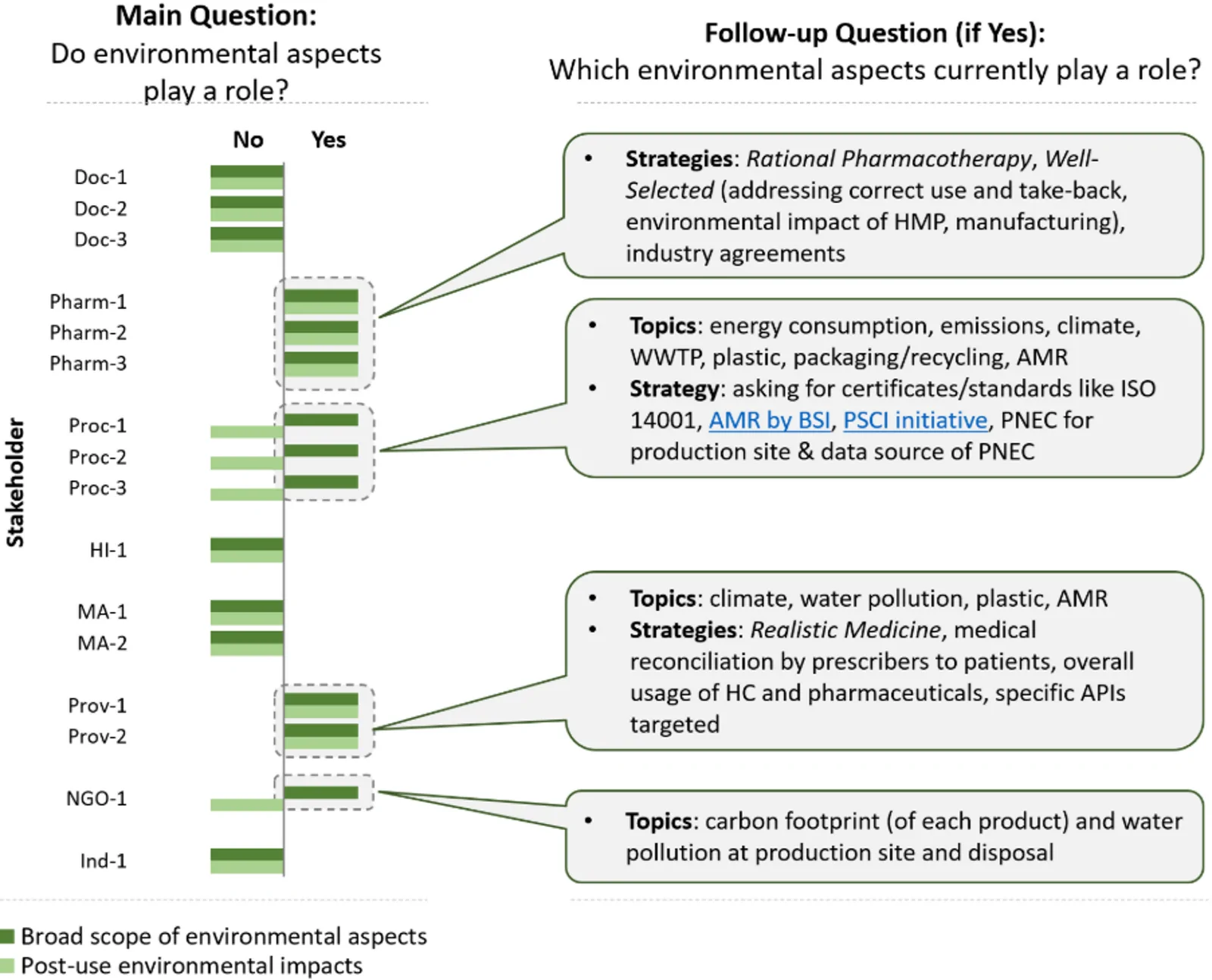
Current and future role of environmental aspects (dark green), including specific post-use environmental impacts (light green), in decision-making of interviewed professionals. Environmental aspects that play a role currently are summarised in the speech bubbles. HMP: human medicinal product; NSAID: non-steroidal anti-inflammatory drug; PiE: pharmaceuticals in the environment; AMR: antimicrobial resistance; BSI: British standards institution [5]; PSCI: pharmaceutical supply chain initiative [40]; PENC: predicted no-effect concentration; HC: healthcare

Seven interviewees stated that environmental aspects do not play a role in their decision-making, although four of them expect that this may change in the future under certain circumstances (Fig. 2). Other interviewees indicated that environmental aspects, including post-use environmental impacts, are accounted for, although the priority areas as well as the policies currently in use (Question 8) varied substantially between the stakeholder groups.

Interviewed pharmacists, procurers and providers (8/8) stated that environmental aspects generally play a role in their decision-making, especially actions concerning climate change, water pollution, plastic/packaging/recycling, and antimicrobial resistance (AMR). Post-use environmental impacts also play a role in the work of pharmacists and providers (5/5), but not for procurers (3/3), although many noted that this may change in future (Fig. 2).

Pharmacists (2/3) emphasized best practices of pharmacies regarding greener pharmaceuticals (also summarised by PGEU, [37]) like the implementation of the environmental symbol *Välvald* in Sweden (in English ‘Well-Selected’) for over-the-counter medicines that meet certain environmental criteria [50]. They also highlighted that pharmacists can only act within an established legal framework: that of dispensing prescriptions meeting the demand of patients for medicines, which then are to be available on demand. All interviewed pharmacists (3/3) implied that pharmacists can inform patients, e.g., about the correct use, environmental risks and about the existence of collection/take-back systems. A key driver for pharmacists’ willingness to support adaptation to environmental safety aspects is increasing demand from patients for information about sustainability of their products and potential alternatives.

Procurers (3/3) reported they have initiated a strategy to incorporate environmental considerations into their tenders, beginning with the availability of environmental information, but that they do not yet take the actual impacts into account. In upcoming tenders, the next step will be to reach an agreement on criteria to be included (still in development). So far, tender invitations by the procurement agencies of the interviewees include questions about energy consumption/emissions/climate, wastewater treatment, plastic, packaging/recycling, questions related to AMR, and whether manufacturers have been certified to standards like ISO 14,001 for Environmental Management Systems.

Providers (2/2) are approaching environmental issues at local and national levels. Examples of this are the development of local recommendations for prescribers considering environmental impacts jointly with conventional parameters (e.g. efficacy, cost), gathering data on the usage and environmental concentrations of certain compounds to provide feedback to prescribers, and cooperating with environmental authorities. A healthcare provision authority is working on a template for clinical guidance on sustainability and the inclusion of post-use environmental impacts into the formulary activities. This interviewee explained that these activities aim to change the mindset of HC professionals, which is considered a win-win for the patient and the environment. Both providers mentioned that in their countries (SE and UK), first steps are being taken at the national level towards prescribing strategies considering environmental aspects, including post-use impacts.

**Fig. 3:**
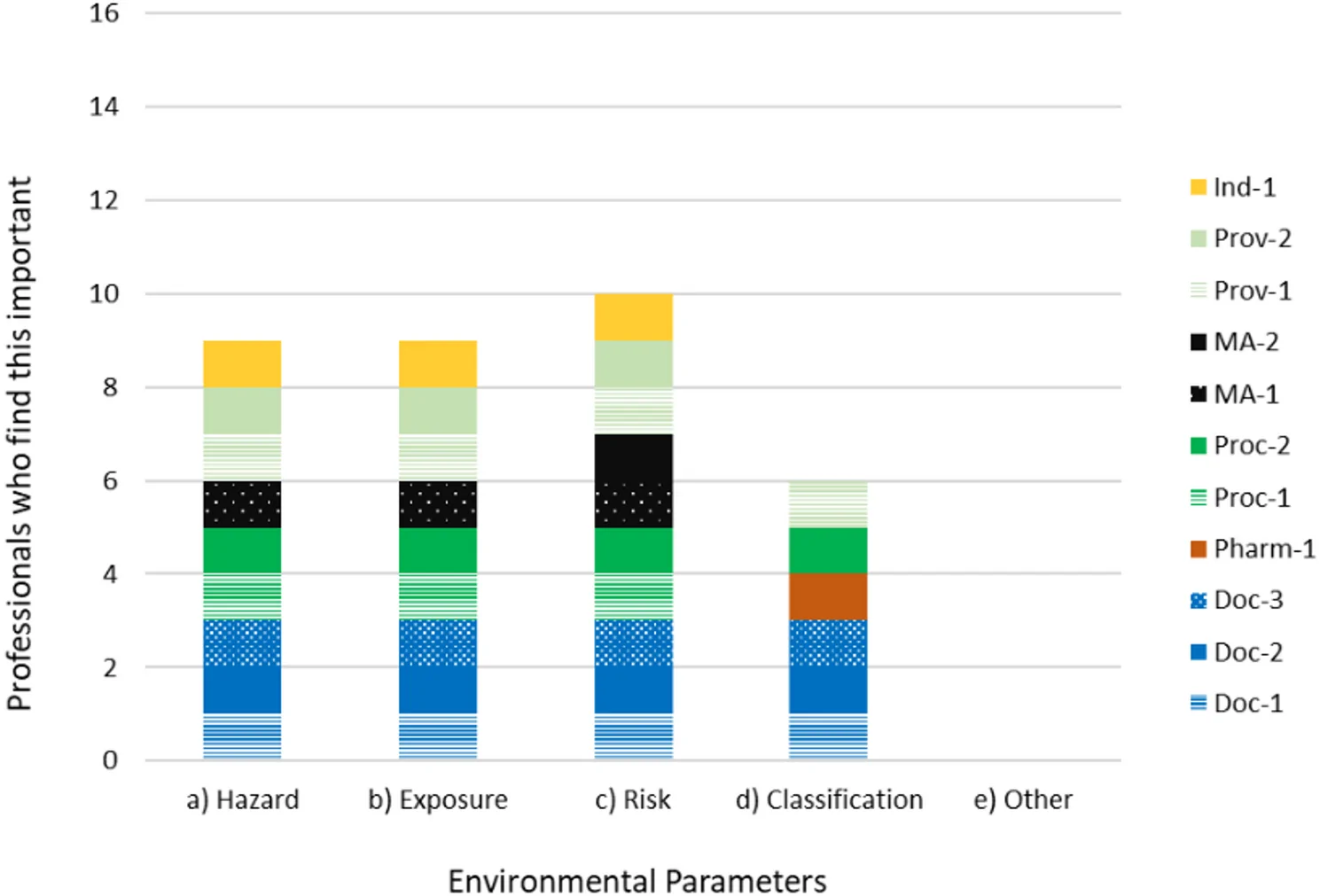
Environmental hazard, exposure and risk (**a-c**) may become relevant in future to take post-use environmental impacts into account. A classification system (**d**) could be developed based on such data to guide environmentally informed decision-making by HC professionals. Interviewees received a list of examples to answer the question which parameters may become relevant: **a**) hazardous properties: PBT, vPvB /PMT, vPvM (footnote 2), **b**) environmental exposure: emissions, environmental concentrations, **c**) environmental risk: PEC/PNEC (footnote 3), **d**) substance classification according to regulation, such as CLP and/or REACH, **e**) other parameters. 5 interviewees (Pharm-2-3, HI-1, NGO-1, Proc-3) did not answer this question due to limited knowledge on post-use environmental impacts

When asked about possible future parameters relevant for post-use environmental impacts *(such as hazardous properties*^2^, *environmental exposure*,* environmental risk*^3^, *substance classification according to regulation (e.g.*,* CLP*^4^*and/or REACH*^5^*regulation)*,* other parameters)*, 10/16 interviewees answered that parameters included in the ERA (hazard, exposure and risk) are important (Fig. 3). One interviewee only mentioned the risk quotient PEC/PNEC.^6^ A classification system is also seen as important by ca. one-third of the interviewees (6/16). This should be based on ERA parameters, understandable, simple, easy to use in daily practice of HC professionals (e.g. traffic light system), but should be set up by others than clinicians, e.g. by authorities. It is noteworthy that 5/16 interviewees did not answer this question due to limited knowledge on post-use environmental impacts.

Interviewees stressed the insufficient understanding of environmental properties, with the lack of reliable, transparent, publicly available data (5/16) as the main theme. Another limitation mentioned specifically for research and development (R&D) is the lack of suitable tools to screen ERA parameters in early R&D (1/16).

#### An environmentally informed selection process for pharmaceuticals: the importance of different criteria

If criteria on post-use environmental impacts become part of future decision-making by HC professionals, these criteria may need to be weighed against other criteria. The interviewees were specifically asked for their views on weighing these criteria in terms of the cost and secure supply of medicines and the patient safety (Question 6; see Table 1 and SI). In addition, they were asked to compare their decision-making logic in different situations (Question 6a).

##### Criteria on costs and secured supply of medicines

Currently, decision criteria for medicines (e.g., for procurement, reimbursement, medical guidelines) are primarily based on efficacy, patient safety, costs, and the security of supply chain. According to several interviewees, environmental impacts are part of these decisions in a few initiatives; and if they are, they have a lower weight than e.g., costs (7/16). Several interviewees mentioned there may be room for compromise (7/16) as often the price for a medicine is not set in stone. This is exemplified e.g. by the Patient Access Scheme (PAS) in Scotland, whereby a pharmaceutical company can propose to improve the cost-effectiveness of a medicine through a cost-reduction mechanism, thus enabling patient access to medicines that would otherwise not be found to be cost-effective by the Scottish Medicines Consortium [32]. Additionally, the room for compromise depends on the economic pressures on the reimbursement system in the given country. For example, four interviewees mentioned the new Joint Nordic Tendering Procedure from Norway, Denmark and Iceland, which uses criteria that weigh 50% for price, 30% for sustainability (not yet including post-use impacts), and 20% for security of supply (see also AMGROS [4]; [33]). Two interviewees felt that this gives a considerably high weight to environmental impacts when compared to the financial resources of their own countries.

##### Criteria on dosing and patient safety associated with increased stability in the environment

Medicines need to be safe and effective, and meet minimal quality standards. Efforts in pharmaceutical R&D are made to minimise safety issues by avoiding adverse drug reactions. This includes avoiding (a) reactive metabolites, (b) transformation by UV-light (risk of phototoxicity), and (c) drug-drug interactions [35], among many other parameters. Ideally, once-daily dosing (d) is pursued to ensure good patient compliance and adherence to a medical treatment. Achieving a good balance of these (a-d) and other parameters may indirectly select for drug substances that are also more stable (i.e., persistent) in the environment due to optimal stability in human.

**Fig. 4:**
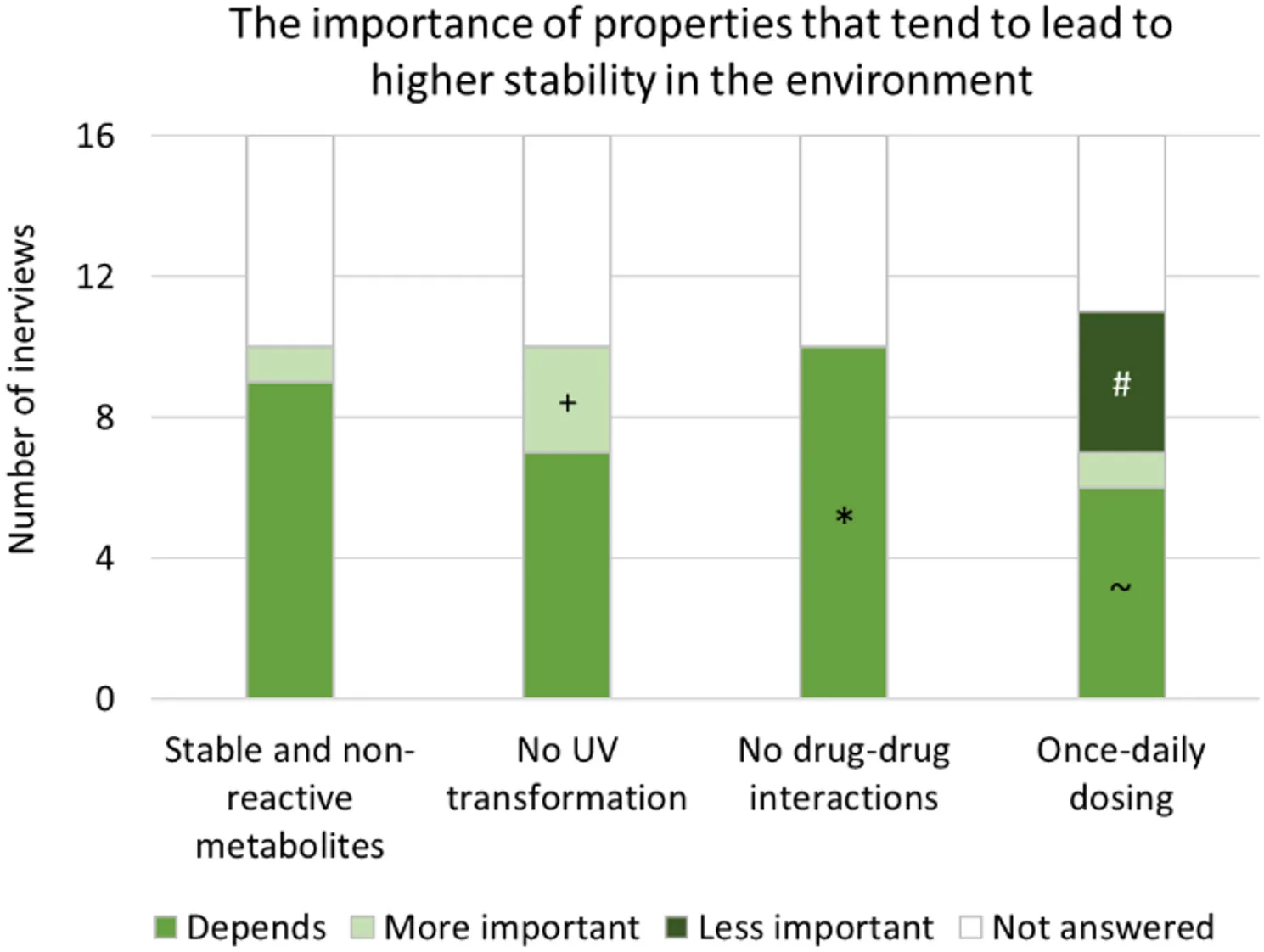
The importance of properties that tend to lead to higher environmental stability. + Risk can be high for some patients; Difficult if medicine need to be hidden from light. * The importance of avoiding drug-drug interactions depends, for example, on the likelihood that these drugs are applied in parallel. ~ Dosing depends also on the patient.# Hospital: Fewer dispensation saves time (i.e., staff) and equipment and allows more ambulant treatment

When asked for the importance of certain requirements for a medicine (related to dosing and patient safety), most interviewees could not provide an answer due to the limits of their mandates or knowledge (5–6/16) or could not provide a straight answer to this question due to many preconditions (6–10/16) (Fig. 4, categories “not answered” and “depends”, respectively). However, some indicated that once-daily dosing could be compromised for environmental benefit (“less important”, 4/16). The dosing also depends on the patient. In the specific case of hospitals, fewer dispensation has the advantage of saving staff time and equipment and allowing more ambulant treatment. In contrast to once-daily dosing, avoiding transformation by UV-light is important (3/16), for example, because of a high risk of phototoxicity for some patients or difficulties if the medicine needs to be hidden from light during workflows in hospitals. According to several interviewees (10/16) the importance of avoiding drug-drug interactions depends, e.g., on which drugs interact, thus on the likelihood that these drugs are applied in parallel.

In summary, interviews suggest that the importance and room for compromise for each of these four aspects may be case specific, depending on the type and seriousness of the disease, and on a balance of benefits and risks, as performed for marketing authorisation. This may also explain why several interviewees not involved in the regulatory risk-benefit analysis felt unable to answer this question. Nevertheless, many interviewees highlighted that patient benefit is a top priority (11/16; referring to drug efficacy and patient safety). A group of patients will accept a greener alternative only if it demonstrates equal patient benefits (NGO-1).

##### Decision-making logic

In the interviews we defined two different scenarios in decision-making, when integrating environmental impacts due to the use of medicines by patients (post-use environmental impacts): *(1) a confirmed post-use environmental impact and (2) no environmental risk despite a hazard classification*. Interviewees mentioned different aspects to be considered, for case *1* as well as *2.* Doctors need to be able to justify their choice for a more environmentally friendly treatment to the patient (1/16). The selection between APIs depends on the full picture, and thus on the level of available information (3/16). This includes, e.g., consumption data, resulting environmental concentrations, the medical need, and level of confidence that there is no risk in case 2. An interviewee remarked that focussing only on those APIs with higher attention or evidence (e.g., diclofenac) could lead to a situation that the risks of other APIs, that may pose a risk too, are overlooked.

For HC procurers and providers (2/5), there is a “can do“ that depends on evidence and policy. Current policies require concrete evidence for harm to human beings to result in actions (e.g., restrictions on use for the pharmaceutical concerned in hospitals), but evidence of human harm from PiE is lacking and harm to the environment itself is not mentioned at all. An interviewee mentioned that for this reason approaches like Realistic Medicine [43] include the precautionary principle through win-win situations for humans and the environment. In addition to the “can do”, there is a preventive “would like to do“ for HC procurers and providers that depends on predictions of the environmental impact potential, i.e., before the effects occur.

According to the interviewee from industry, regulatory pressure and pressure from environmentally conscious patients could drive the uptake of environmental considerations, which could be based on flags, e.g., related to PBT criteria. This could move towards a “best-in-class” approach.

#### Potential for any unintended consequences

Including criteria on post-use environmental impacts into the decision-making by HC professionals requires an understanding of possible unintended consequences. When asked about such consequences and related mitigation measures (Question 7, see Table 1), six main scenarios were identified by interviewees (Table 3).

**Table 3:** Unwanted consequences and their mitigation measures brought up by the interviewees

Unwanted consequence	Corresponding mitigation measure
Legal dispute between stakeholders (e.g., when an industry agreement is attempted by pharmacies, but industry is referring to the need for access to medicines),	
Trade-offs with other environmental impacts (e.g., lower post-use environmental impact, but higher carbon footprint),	Sufficient and transparent data on trade-offs to understand the full picture.
Clinicians feeling overwhelmed or adhering to familiar treatments (i.e., continuing to use drugs that they have always relied on),	Clear guidance for HC professionals by authorities/associations.
Patients choosing not to switch to the new drug (e.g., if new drug is less effective or takes longer to work),	Education of patients and wider public.
Supply shortages and increasing dependence on other countries (e.g., when stricter requirements are imposed in one country compared to others),	Harmonised regulations on EU level as this creates more market power than initiatives on national level.
Greenwashing.	Clear definition of “green(er)”.

Some possible unintended consequences were mentioned that are independent of the addition of environmental criteria, for example risks for the patient (risks for side-effects), closure of production sites resulting in supply bottlenecks, and oversupply of an API that was deselected suddenly. Four interviewees argued that there are no additional risks for the patient when selecting a greener API because pharmaceuticals always need to be effective and safe, which is ensured by the MA process.

### Section 3 - Barriers and needs to introducing environmental considerations in decision-making of HC professionals

#### Needs to be addressed and actions required to account for post-use environmental impacts in decision-making

When asked about the needs to be addressed and actions required to incorporate environmental considerations into decision-making processes along the pharmaceutical life cycle (Question 9, see Table 1), three main themes were identified:

##### Open availability of reliable data

The majority expressed a need for reliable and accessible scientific evidence and data on environmental impacts (9/16). The importance of scientific evidence as a foundation for subsequent regulatory obligations was emphasised (8/16). It was also stated that regulatory ERA data should be (better) publicly available and the industry should provide more data (3/16). An information and classification system such as the one that already exists in Sweden (Fass)^7^ was mentioned as one possibility for providing data (2/16).

Transparency was another recurring theme (7/16). Pharmacists and professionals from procurement, provision and NGO called for greater transparency and publicly available data on pharmaceutical production and post-use environmental impacts.

##### Legislative/regulatory actions

Besides this, there was a strong call for legislation and regulatory frameworks (13/16). Two interviewees saw legal obligations as a necessary follow-up to scientific evidence for environmental impacts by PiE. According to a procurement professional, a regulatory framework is needed to take environmental criteria into account in procurement decisions and to deprioritise price criteria. The interviewee working at a health insurance company also stressed the importance of including sustainability aspects in regulatory frameworks, as health insurance companies only have limited leeway within their legal mandate. Likewise, according to another interviewee, marketing authorisation authorities need legislation and guidelines to be able to evaluate pharmaceuticals based on environmental criteria. The interviewee from industry said that a harmonised regulatory framework would be needed to support the market success of environmentally friendly products, while taking into account the therapeutic benefit. In their view, the transition times for such a framework would need to consider the long timelines of pharmaceutical R&D (see for example [30]).

##### Harmonised guidance and education

Collaboration and harmonisation were frequently mentioned (8/16). One of the pharmacists suggested harmonising guidelines and (best) practices across Europe. Procurement professionals (2/3) emphasised the need for collaboration and harmonisation, particularly in regulatory frameworks and data sharing, to effectively address environmental concerns. Proc-3 stressed that a common way of weighing and discussing the environmental impact is needed.

Lastly, education and awareness emerged as critical components (9/16). Continuous education for HC professionals to raise their awareness of this topic were emphasised (2/16). Also, the importance of a simple and easy-to-use framework provided to prescribers was highlighted, with reference made to the Swedish Wise List (3/16).

There was broad agreement between most interviewees on the essential expertise needed to assess and weigh the various types of API criteria. It was highlighted that an interdisciplinary approach is crucial, which involves combining traditional pharmaceutical knowledge with environmental science (especially ecotoxicology) and incorporating societal and health-economic expertise. For instance, NGO-1 advocated for interdisciplinary work to ensure that all relevant aspects, from human health to ecosystem impacts and financial considerations, are addressed. It was noted by a market authorisation professional that efficacy and safety are already addressed in the marketing authorisation process, with sufficient expertise available in these areas. Another market authorisation professional mentioned that although ecotoxicological expertise exists in their organisation, they are lacking in personnel capacity.

#### Potential roles of stakeholders in considering environmental impacts

When asked about the expertise needed for performing a holistic assessment across efficacy, safety as well as post-use environmental effects of pharmaceuticals (Question 10, see Table 1), three main themes emerged.

##### Collaborative effort

All but one interview partner (15/16) agreed on the necessity of collaboration among various types of authorities (MA, environmental), HC professionals, industry, and procurement agencies. Prov-1 highlighted the need to build a web of information considering different dimensions from different perspectives. According to Proc-3, HC actors should be involved to validate results and decisions that build upon that, and to increase the acceptance thereof. However, another point underlined was that doctors and pharmacists lack time and often also knowledge to take environmental criteria into account (4/16).

##### Key stakeholders

Authorities were rated as the most important actors to make this holistic assessment by all groups of interviewees (13/16). The marketing authorisation process is seen as the biggest lever with an impact on many other areas (NGO-1). A pharmacist stated that assessment and weighing should be conducted at the level where decisions are made regarding which pharmaceuticals are introduced to the market. There were differences in opinion on whether MA authorities alone should conduct assessments or if other stakeholder groups (e.g., environmental authorities, third parties) should lead or be involved. For instance, if there is a lack of expertise on the topic in the MA authority, (parts of) the assessment could be outsourced to environmental authorities or public research institutes (MA-1). The importance of independent third parties, to obtain independent assessments, was emphasised (Pharm-3). It was acknowledged by two interviewees that the pharmaceutical industry has a key role in providing essential data but there are concerns about conflict of interest, transparency and data reliability.

##### High level guidance

It was stressed by two interviewees that the holistic assessment should be performed according to guidelines at the highest level possible, preferably by EMA or a European health technology assessment body [10], to enable the greatest possible harmonisation in decision-making processes. If national/regional bodies were to do it at their respective level, there would be duplication of effort. Diverging processes and methodologies could jeopardise the ‘level playing field’ and lead to access inequities. National/regional organisations could use the central assessment outcomes to prioritise drugs within specific HC areas (Prov-2). Nevertheless, it was emphasised by three interviewees that there may be differences for different HC systems, e.g. insurance-based systems, or national public health services. Therefore, national HC actors and authorities would need to work together to ensure harmonisation.

## Discussion

### Characteristics of the interviewees and strengths and limitations of the study design

The selected various professionals covered the whole pharmaceutical lifecycle, resulting in different perspectives on the significance of environmental considerations (Figs. 1 and 2). For example, the results show that the preparedness to include environmental considerations in their decision-making processes depends on the specific background of the interviewee. Studying different perspectives corresponds to the study approach with a heterogenous sample and its aim of drawing initial conclusions about these various perspectives.

We prioritised selecting interviewees with different roles in the lifecycle above selecting interviewees that represent all EU countries. Interviewees that rejected the invitation or did not respond were mostly (17/25) not immediately affiliated with the PREMIER members’ network, which may be indicative of their unfamiliarity with the topic. Because of this, selection bias may have arisen from the fact that interviewees represent countries that already have some form of policy on pharmaceuticals in the environment and already have some knowledge on the subject. This bias therefore inevitably affects the generalisability of our findings.

The study faced challenges in reaching representatives from the stakeholder group “society representatives / patient organisation”. Despite reaching out to eight individuals from this group, none accepted the interview request (Table 2). In addition, the views of EU level decision-making bodies and authorities and those of national pharmaceuticals pricing boards could not be collected in this study, although these stakeholder groups too can have critical roles in terms of implementing incentives toward adaptation of greener APIs.

Interviews with professionals are a well-established qualitative research method, valued for their ability to provide in-depth insights from individuals with specialised knowledge [57]. The objective of this interview study was not to achieve statistical representativeness, but rather to explore complex issues in detail, drawing on the nuanced perspectives of informed participants.

For two reasons, the extrapolation of the outcomes to the different stakeholder groups addressed is hindered: (1) The limited number of interviewees included (*n* = 16) and the inherent biases in their selection. (2) Interviews were conducted with people that are already interested and active in this field and thus are willing to spend their time. Therefore, the outcomes need to be taken as individual opinions that can used to identify concerns and opportunities that enable uptake of post-use environmental impacts in the decision-making process. Each of these professionals are experts in their own field but might not have sufficient knowledge on environmental aspects to integrate all necessary considerations to address some of the questions. Hence, the conclusions drawn should be assessed critically and results interpreted with caution, especially when making broader claims about the subject. In this way, the results contribute to understanding this complex topic.

Another aspect of the interviewee selection is the overrepresentation of interviewees from Nordic countries (8/16 participants being based in SE, NO, FI and DK). This skew in geographical distribution likely reflects the composition of the PREMIER members’ network, which is concentrated in North-western Europe. While this geographical focus has undoubtedly influenced the perspectives and experiences shared in the interviews, it was carefully considered during the interpretation of the results. Efforts were made to account for this bias by contextualising findings within the broader European framework and acknowledging that certain trends (e.g., more environmental strategies by pharmacies and procurement agencies) or insights may be more reflective of Nordic-specific conditions.

### Significance of environmental aspects at present and in future

The results on the current role of environmental aspects in decision-making (with broader scope of environmental sustainability, including carbon footprints, and not exclusively focused on post-use) show that these aspects are included to a certain extent in some initiatives. There are limits due to professional’s mandate and level of knowledge. Sometimes there is a specific focus, e.g., on environmental issues related to production (e.g., energy consumption, emissions; see Fig. 2 right side), which reflects the current focus in the pharmaceutical sector and wider society [1, 6]. Currently, post-use environmental impacts play a minor role (Fig. 2). This finding is also in line with the interview study by Riikonen et al. [44] with pharmaceutical companies represented in Finland, showing that ‘improving waste management’ and ‘reducing impacts of their own operations’ is already taking place within the companies, whereas ‘environmental impacts arising from drug consumption’ was mentioned frequently as one of the challenges difficult to resolve due to its complexity and the need of multi-stakeholder collaboration.

Interviewees expressed an expectation that PiE may play a role in their decision-making in future if the awareness and knowledge of this topic continue to grow and the policy framework changes (Fig. 2). There is now increasingly more activity in this field, e.g. linked to the ‘Pharmaceutical Strategy for Europe’ [11] and the revision of the pharmaceutical legislation [13, 17]. Nevertheless, it is not clear at this stage how exactly the policy framework needs to change. The TransPharm project (2022–2026) aims to provide a perspective on future possibilities on the actual use of greener pharmaceuticals, including the chances for a business case for greener pharmaceuticals.

The interviewees considered environmental parameters that are related to the regulatory ERA to be important for an environmentally informed decision-making. This can be explained by professionals being familiar with this data or having at least heard about it. Using the data or results of regulatory ERAs would allow for a harmonised assessment of environmental impacts, not only by pharmaceutical companies and authorities responsible for MA but also by other actors involved in HC.

For the inclusion of ERA parameters into the decision-making process, there may be different approaches to weigh them against other criteria, depending on professional’s expertise and mandate, and whether the weighing is before or after MA. For example, we conclude from the interviews that procurement agencies and HI companies weigh the price quite high, while doctors prescribe and give advice in a patient-centric manner. In addition, the interviewees’ answers showed that balancing properties related to patient benefit and risk against environmental properties could be a case-by-case decision. Three generic prerequisites for this decision could be identified: it needs to be patient-centred, evidence based, and transparent. In addition, an evidence-based decision would require a thorough understanding of the environmental impacts to be considered, which is currently not the case with all HC professionals. Thus, it could also be argued that decisions should be made on a higher level (e.g., by guideline writers) and not by doctors themselves.

The patient-centred criterion is in line with another interview series, where interviewees stated that a choice for a greener treatment option can only be made if patient benefit, safety and compliance are not compromised [19]. Cohen et al. [8] advocate for integrating individual considerations, societal responsibilities and systemic changes to promote environmentally sustainable healthcare. The reasoning from Cohen et al. [8] is based on the recognition of the environmental crises as a health crisis. Hence, the importance of the patient-centred criterion is an ethical question, and this debate is still in its infancy [23]. Kuiter et al., performed a systematic review of reasons for and against including environmental aspects in the decision-making of HC professionals. They shed light on a possible transformation of bioethical principles.

### Barriers and needs to introducing environmental considerations in decision-making

#### Lack of data and knowledge: The need for a database and collaboration between disciplines

The needs for transparency and accessibility of scientific data that informs on post-use environmental impacts of medicines, as expressed by the interviewees, echo similar calls by other actors such as academia and environmental authorities (see, for example, [2, 17, 25, 34, 38] A significant amount of data already exists, but much of it is either difficult to find and access or not publicly available at all [17]. A lot of relevant data is included in the ERAs submitted during the marketing authorisation process and should be published in Public Assessment Reports (PARs) and European Public Assessment Reports (EPARs). However, depending on the rapporteur Member State, these documents are not always easy to find or access and the quality of the published information varies considerably [17]. Within the EU PREMIER project, a database with environmental information is currently being built for those pharmaceuticals for which data is available. Even though they were not directly asked, interviewees expressed a clear interest in such a database, especially if it would be hosted by a regulatory authority.

In spite of the fact that more data (e.g., on environmental fate and effects) is needed, a significant amount of expertise in ERA and knowledge in ecotoxicology already exists in pharmaceutical companies and authorities (Section “Characteristics of the interviewees and strengths and limitations of the study design”). Interviewees expressed support for an interdisciplinary approach to enable a transfer of knowledge and expertise, for example in ecotoxicology.

#### Lack of time and expertise: The need of an information & classification system

The perceived level of knowledge of the various professionals corresponds to their potential roles. Interviewed doctors and pharmacists consider themselves as having neither the time nor the expertise to interpret data on environmental effects and exposure or to perform an ERA by themselves. This is in line with a survey with 290 medical, nursing, and physician assistant students at Yale university. The survey showed that, next to missing education in environmental issues, lack of time and productivity pressure are relevant barriers for HC professionals to take responsibility for the environment [47]. However, the role of doctors, pharmacists and similar stakeholder groups should be neither overestimated nor neglected, as they are in direct contact with the patients.

The interviewed HC professionals asked for an information and classification system for environmental risks and hazards that is objective, easy-to-use and transparent. Such a system can then be used to identify for which APIs risk mitigation measures should be taken. In Sweden, there are already two knowledge supports in use, Janusinfo and FASS. Janusinfo builds on the data from FASS but also includes data from industry that they used for marketing authorisation procedures and external studies. Janusinfo is generally preferred by stakeholders (e.g., pharmaceutical industry, authorities) because of its transparency, the presentation per API and the use of information in clinical practice [25]. However, it focuses on the actual use in Sweden, leading to risk estimates for the Swedish situation, and thus cannot simply be translated to other countries. Moreover, using the Swedish systems is challenging, for example in terms of lack of data and difficulties considering the environmental aspect of pharmaceuticals in HC practice. Respondents wanted more knowledge, clear messages, and legislation to support their work to reduce the negative environmental impact of pharmaceuticals. Nevertheless, the systems could inspire the development of a classification system that is harmonised for use in other countries [26, 42].

National HC systems and national/regional conditions of medicine use can vary significantly, necessitating a tailored assessment due to different use patterns. However, the assessment methodology should be harmonized to avoid potential inequities or discrimination. Thus, there is a call for a centralised and harmonised approach towards including environmental impact in decision-making by HC professionals, which needs participation at various levels to also allow region-specific aspects. This trend is consistent with the findings of Heni et al., [19], which examined user requirements concerning sustainability assessments of pharmaceuticals.

#### The MA process as a central intervention point

Authorities were rated by interviewees across all stakeholder groups (13/16) as the most important stakeholder for considering environmental aspects, while pharmaceutical companies were mentioned in particular with regard to their role as the originator of environmental safety data. Currently, regulatory ERAs are performed by pharmaceutical companies (in connection with marketing authorisation applications and according to the relevant guideline), which is then reviewed by authorities. Expert knowledge of the underlying data therefore resides with the responsible departments of both stakeholder groups. This may not be the same for the task of weighing between the wider criteria (safety, efficacy, cost – and broader environmental sustainability aspects such as carbon footprints) or choosing between products, which is a challenging task. A team of different experts would be required to analyse a wealth of complex and not always comparable data. Such intensive evaluations would lead to additional costs, an issue that to the best of authors’ knowledge has not yet been investigated.

Full representation of all stakeholder groups in the decision-making process and full transparency of the decisions made would generate trust and buy-in across the stakeholder landscape and mitigate against the risk of any decisions resulting in unintended consequences.

While the established regulatory ERA process already generates data on the environmental impacts of pharmaceuticals, there remains room for improvement (e.g., [17]). Another challenge lies in the need to compare between APIs or between medicinal products (with the same or different APIs). This raises several unresolved questions, such as which criteria with which indicators should be used, how these criteria should be weighted or aggregated, and what decisions can and should be drawn from these comparisons (discussed by van Wilder et al. [55]). Additionally, interviewees expressed a strong desire for centralisation and harmonisation, not only regarding data (see above) but also regarding the underlying methodological aspects and the way weighting could be applied. In this context, the EMA or an EU Health-Technology-Assessment body was mentioned several times as a suitable option.

As the European MA process has been identified as a central intervention point, it is important to investigate any possibilities within this framework. Conclusions on environmental risks of human medicinal products are not currently part of the risk-benefit analysis. The draft new pharmaceutical legislation includes the possibility to refuse marketing authorisation approval if “…the environmental risk assessment is incomplete or insufficiently substantiated by the applicant or if the risks identified in the environmental risk assessment have not been sufficiently addressed” (Art. 47, directive proposal, [13]). Other considerations, e.g. considering undesirable environmental effects in the risk-benefit analysis, are described by Gildemeister et al. [17].

#### Identified unintended consequences

Interviewees identified six possible unintended consequences which could happen when including environmental criteria into decision-making, and proposed potential solutions to mitigate them (Table 3). Concerns were raised that greener APIs may threaten access to medicines. Trade-offs between the different impacts on environmental sustainability were also mentioned (this point is discussed at length by Moermond et al. [28]. Additionally, possible trade-offs with economic and social sustainability were discussed by the interviewees. For example, supply shortage in one country may be caused by stricter requirements than in other countries.

While harmonised regulations and methodologies on the EU level (mentioned as a solution) may avoid that, there needs to be room for decisions about the use of medicines at a national or regional level. Ideally, this could be in accordance with the principle of subsidiarity (e.g., in some cases, prescribers could select medicines that are less harmful to the environment in situations where there are several alternatives that provide equivalent treatment using provided tools or guidelines). This approach includes education and awareness-raising among HC professionals, integrating environmental criteria into formularies, standards, guidelines, and regulatory frameworks, and incorporating diverse interdisciplinary expertise. Such a multidimensional approach is e.g. also suggested by Gad et al. [16] and Alejandre et al. [3].

#### An overarching need: regulatory changes

Regulatory changes are essential to integrate post-use environmental impacts into decision-making, as reflected in the interviewees’ responses. These changes are not only needed at the European level but also at a national level. Regulatory frameworks define the scope of action for many actors in the HC system. Revisions are required to enable authorities responsible for MA, health insurance companies, procurement agencies, and HC professionals to integrate environmental considerations more strongly into their decision-making processes. Once regulatory frameworks and funding mechanisms mandate or incentivise greener API development, research and clinical practice typically adapt accordingly.

## Conclusion

This study describes results of in-depth interviews with 16 professionals of eight healthcare stakeholder groups, such as procurers, prescribers and pharmacists, and contributes to the scientific discourse on societal implementation of APIs with reduced environmental impact.

The findings inform on the current views of health care professionals regarding minimising environmental impacts of medicine by using greener APIs in the health care sector. Holistic assessment systems with different criteria are needed to make decisions. Increasing willingness by HC professionals to consider environmental parameters may stimulate data sharing in the short-term and, in the long-term, may also stimulate development of tools and assays to design greener APIs. Incorporating environmental impact criteria in decision-making along the pharmaceutical lifecycle –is a prerequisite for change. This should specifically include the marketing authorisation process, which was identified as a possible point of intervention.

For such a change to happen, environmental criteria with their thresholds that can be used in drug discovery processes need to be defined, and reliable in silico models and in vitro assays with adequate throughput need to be developed. However, there is currently no clear business case to develop greener APIs that would motivate pharmaceutical companies to include post-use environmental criteria into their R&D paradigm. Future research is needed to investigate the interconnectivity between the social, environmental and economic benefits and risks of the development of greener APIs.

This interview study identified needs to be met and barriers to overcome to implement environmental considerations into decision-making by HC professionals. There is a need for reliable scientific data and evidence of environmental impacts, multidisciplinary cross-stakeholder discourse and collaboration, legislative change and a revised regulatory framework that includes a harmonised approach to include environmental criteria in the decision-making by HC professionals.

We propose a stepwise approach to harmonise the uptake of greener APIs while allowing for region-specific features:

1. As a first assessment step, the API-inherent physico-chemical and ecotoxicological properties should be assessed. This also allows comparisons of APIs when risk assessment is performed based on use per patient per day for a certain indication. Results could be made publicly available, e.g., in the format of a monograph system, as it is now also suggested in the new pharmaceutical legislation [13]
2. The second assessment step could take place at a national or regional level to identify the need for mitigation measures in case of an identified risk on a national or regional level. This assessment should include specific conditions of medicine use and other regional-specific features.

## Supplementary Information

Below is the link to the electronic supplementary material.

Supplementary Material 1

## Data Availability

All data supporting the findings of this study are available within the paper and its Supplementary Information. Data from semi-structured interviews cannot be publicly available due to privacy and confidentiality concerns. For enquiries about accessing the data, please contact Dr Oliver Olsson, email address: oliver.olsson@leuphana.de.

## Footnotes

1 Novel entities: “new substances, new forms of existing substances and modified life forms”, including “chemicals and other new types of engineered materials or organisms not previously known to the Earth system as well as naturally occurring elements (for example, heavy metals) mobilised by anthropogenic activities” [49].

2 Hazardous APIs are APIs that are persistent, bioaccumulative and toxic (PBT), very persistent and very bioaccumulative (vPvB), persistent, mobile and toxic (PMT), or very persistent and very mobile (vPvM).

3 Defined by the risk quotient PEC/PNEC (PEC: predicted environmental concentration; PNEC: predicted no-effect concentration).

4 Regulation (EC) No 1272/2008 on classification, labelling and packaging of substances and mixtures.

5 Regulation (EC) No 1907/2006 concerning the Registration, Evaluation, Authorisation and Restriction of Chemicals (REACH), establishing a European Chemicals Agency (ECHA).

6 See footnote [3].

7 Fass.se (Fass) is Sweden’s national formulary. On Fass, under the “miljöinfo” tab for a pharmaceutical product, environmental risk, degradation, and bioaccumulation are presented (in Swedish). Detailed data is also provided. Environmental data on Fass is provided by industry voluntarily and is based on companies’ own studies or peer-reviewed literature. Before the data is included in Fass, it is reviewed by the IVL, Swedish Environmental Research Institute. The associated guidance can be found here: https://www.lif.se/contentassets/b7cf255755504f78a906f3eba8a6ae38/environmental-classification-of-pharmaceuticals-att-wwwfassse.pdf.

## References

[1] ACS GCI Pharmaceutical Roundtable. Focus areas. https://acsgcipr.org/focus-areas/. (retrieved 30.06.25).

[2] Ågerstrand M, Berg C, Björlenius B, Breitholtz M, Brunström B, Fick J, Gunnarsson L, Larsson DGJ, Sumpter JP, Tysklind M, Rudén C. Improving Environmental Risk Assessment of Human Pharmaceuticals. Environ Sci Technol. 2015;49(9):5336–45. 10.1021/acs.est.5b00302. 25844810

[3] Alejandre J, Frascaroli G, Escudero A, Pahl O, Price L, Pfleger S, Helwig K. informed pharmaceutical prescribing in Scotland: current policy landscape and proposed policy options to enable the implementation of eco-directed pharmaceutical prescribing in the Scottish healthcare system: CREW Policy Brief: CRW2020_19 CREW Policy Fellowships Programme. Scotland’s Centre of Expertise for Waters (CREW). 2022. https://www.crew.ac.uk/sites/www.crew.ac.uk/files/publication/CRW2020_19%20Environmentally%20informed%20pharmaceutical%20prescribing-CPF%20Policy%20Brief%20vFINAL%2020220330.pdf. (retrieved 30.06.25).

[4] AMGROS. 2024. Suppliers strongly support joint Nordic tendering procedures. https://amgros.dk/about-amgros/news/suppliers-strongly-support-joint-nordic-tendering-procedures/. (retrieved 30.06.25).

[5] AMR Industry Alliance Antibiotic Manufacturing Standard. 2022. https://www.amrindustryalliance.org/antibiotic-manufacturing-standard/. (retrieved 30.06.25).

[6] Ang KL, Saw ET, He W, Dong X, Ramakrishna S. Sustainability framework for pharmaceutical manufacturing (PM): A review of research landscape and implementation barriers for circular economy transition. J Clean Prod. 2021;280:124264. 10.1016/j.jclepro.2020.124264.

[7] aus der Beek T, Weber F-A, Bergmann A, Grüttner G, Carius A. Pharmaceuticals in the environment: global occurrence and potential cooperative action under the Strategic approach to international chemicals management (SAICM): Texte | 67/2016. 2016. https://www.umweltbundesamt.de/en/publikationen/pharmaceuticals-in-the-environment-global. (retrieved 30.06.25).

[8] Cohen ES, Kringos DS, Hehenkamp WJK, Richie C. Harmonising green informed consent with autonomous clinical decision-making: a reply to Resnik and Pugh. J Med Ethics. 2024;50(7):498. 10.1136/jme-2024-109863. 38290854

[9] EEA. Managing the systemic use of chemicals in Europe. 2023. https://www.eea.europa.eu/publications/managing-the-systemic-use-of (retrieved 30.06.25).

[10] EMA. 2025. Health technology assessment bodies. https://www.ema.europa.eu/en/partners-networks/health-technology-assessment-bodies. (retrieved 30.06.25).

[11] European Commission. 2019. European Union Strategic Approach to Pharmaceuticals in the Environment. https://environment.ec.europa.eu/topics/water/surface-water_en. (retrieved 30.06.25).

[12] European Commission. 2020. Chemicals Strategy for Sustainability Towards a Toxic-Free Environment. https://environment.ec.europa.eu/strategy/chemicals-strategy_en. (retrieved 30.06.25).

[13] European Commission. Reform of the EU pharmaceutical legislation. 2023 https://health.ec.europa.eu/medicinal-products/legal-framework-governing-medicinal-products-human-use-eu/reform-eu-pharmaceutical-legislation_en. (retrieved 30.06.25).

[14] Finckh S, Beckers L-M, Busch W, Carmona E, Dulio V, Kramer L, Krauss M, Posthuma L, Schulze T, Slootweg J, von der Ohe PC, Brack W. A risk based assessment approach for chemical mixtures from wastewater treatment plant effluents. Environ Int. 2022;164:107234. 10.1016/j.envint.2022.107234. 35483182

[15] Foley G, Timonen V, Conlon C, O’Dare CE. Interviewing as a Vehicle for Theoretical Sampling in Grounded Theory. Int J Qualitative Methods. 2021;20:1609406920980957. 10.1177/1609406920980957.

[16] Gad M, Salem A, Oortwijn W, Hill R, Godman B. Mapping of Current Obstacles for Rationalizing Use of Medicines (CORUM) in Europe: Current Situation and Potential Solutions. Front Pharmacol. 2020;11:144. 10.3389/fphar.2020.00144. 32194401 PMC7063972

[17] Gildemeister D, Moermond CTA, Berg C, Bergstrom U, Bielská L, Evandri MG, Franceschin M, Kolar B, Montforts MHMM, Vaculik C. Improving the regulatory environmental risk assessment of human pharmaceuticals: Required changes in the new legislation. Regul Toxicol Pharmacol. 2023;142:105437. 10.1016/j.yrtph.2023.105437. 37354938

[18] Gray LM, Wong-Wylie G, Rempel GR, Cook K. Expanding qualitative research interviewing strategies: Zoom video communications. Qualitative Rep. 2020;25(5):1292–301. 10.46743/2160-3715/2020.4212.

[19] Heni Y, Vidaurre R, Rechlin A, Araujo A. User requirements concerning sustainability assessments of pharmaceuticals: D4.1 - Stakeholder views, needs & wishes on sustainability assessment of APIs. 2023. https://transforming-pharma.eu/sites/default/files/documents/Attachment_D4.1.pdf. (retrieved 30.06.25).

[20] Hester RE, Harrison RM. Pharmaceuticals in the Environment. Royal Society of Chemistry; 2015.

[21] Holdgate MW. A Perspective of Environmental Pollution. Environ Conserv. 1981;8(1):84. 10.1017/S0376892900026989.

[22] Hsieh H-F, Shannon SE. Three Approaches to Qualitative Content Analysis. Qual Health Res. 2005;15(9):1277–88. 10.1177/1049732305276687. 16204405

[23] Kuiter SG, Herrmann A, Mertz M, Quitmann C, Salloch S. Should healthcare professionals include aspects of environmental sustainability in clinical decision-making? A systematic review of reasons. BMC Med Ethics. 2025;26(1):78. 10.1186/s12910-025-01230-4. 40611029 PMC12226885

[24] Kümmerer K. Sustainable from the very beginning: rational design of molecules by life cycle engineering as an important approach for green pharmacy and green chemistry. Green Chem. 2007;9(8):899–907. 10.1039/B618298B.

[25] Linder E, Villén J, Nekoro M, Wettermark B, Sporrong SK. Stakeholders’ perspectives and use of web-based knowledge support for environmental information on pharmaceuticals. Exploratory Res Clin Social Pharm. 2023a;11:100303. 10.1016/j.rcsop.2023.100303. PMC10387601 37529031

[26] Linder E, Wettermark B, Ovesjö M-L, Sporrong SK, Ramström H. Knowledge support for environmental information on pharmaceuticals: experiences among Swedish Drug and Therapeutics Committees. BMC Health Serv Res. 2023b;23(1):618. 10.1186/s12913-023-09646-7. 37309002 PMC10259041

[27] Moermond CTA, Puhlmann N, Brown AR, Owen SF, Ryan J, Snape J, Venhuis BJ, Kümmerer K. GREENER Pharmaceuticals for More Sustainable Healthcare. Environ Sci Technol Lett. 2022;9(9):699–705. 10.1021/acs.estlett.2c00446. 36118957 PMC9476650

[28] Moermond CTA, Puhlmann N, Pieters L, Matharu A, Boone L, Dobbelaere M, Proquin H, Kümmerer K, Ragas AMJ, Vidaurre R, Venhuis B, Smedt Dd. Eco-pharma dilemma: Navigating environmental sustainability trade-offs within the lifecycle of pharmaceuticals – A comment. Sustainable Chem Pharm. 2025;43:101893. 10.1016/j.scp.2024.101893.

[29] Moermond CTA, de Rooy M. The Dutch chain approach on pharmaceuticals in water: Stakeholders acting together to reduce the environmental impact of pharmaceuticals. BMC Health Serv Res. 2022;88(12):5074–82. 10.1111/bcp.15509. PMC9826305 36000992

[30] Möhrle JJ. How long does it take to develop a new drug? Lancet Reg Health – Europe. 2024;43. 10.1016/j.lanepe.2024.100998. PMC11283072 39070760

[31] Monforte J, Úbeda-Colomer J. Tinkering with the two-to-one interview: Reflections on the use of two interviewers in qualitative constructionist inquiry. Methods Psychol. 2021;5:100082. 10.1016/j.metip.2021.100082.

[32] Scottland NHS. Patient Access Schemes. 2020. https://scottishmedicines.org.uk/making-a-submission/companies/patient-access-schemes/. (retrieved 30.06.25).

[33] Nordic Pharmaceutical Forum. International standard against resistance to antibiotics included in Nordic tendering procedures. 2024. https://nordicpharmaceuticalforum.com/index.php/2024/03/11/international-standard-against-resistance-to-antibiotics-included-in-nordic-tendering-procedures/ (retrieved 30.06.25).

[34] OECD. 2019. Pharmaceutical Residues in Freshwater. https://www.oecd.org/en/publications/pharmaceutical-residues-in-freshwater_c936f42d-en.html (retrieved 30.06.25).

[35] Palleria C, Di Paolo A, Giofrè C, Caglioti C, Leuzzi G, Siniscalchi A, Sarro Gd, Gallelli L. Pharmacokinetic drug-drug interaction and their implication in clinical management. J Res Med sciences: official J Isfahan Univ Med Sci. 2013;18(7):601–10. PMC3897029 24516494

[36] Persson L, Carney Almroth BM, Collins CD, Cornell S, Wit CAd, Diamond ML, Fantke P, Hassellöv M, MacLeod M, Ryberg MW, Søgaard Jørgensen P, Villarrubia-Gómez P, Wang Z, Hauschild MZ. Outside the Safe Operating Space of the Planetary Boundary for Novel Entities. Environ Sci Technol. 2022;56(3):1510–21. 10.1021/acs.est.1c04158. 35038861 PMC8811958

[37] PGEU. 2022. PGEU Best Practice Paper on Green and Sustainable Pharmacy in Europe: Pharmaceutical Group of European Union. https://www.pgeu.eu/publications/pgeu-best-practice-paper-on-green-and-sustainable-pharmacy-in-europe/. (retrieved 30.06.25).

[38] Piët JD, Booth A, Donker EM, de Ponti F, Lunghi C, Poluzzi E, Janssen BJA, Tun S, Bekker C, Dima L, Costa J, Jalving M, Munnink O, van den Bemt TH, Patricia MLA, Labriffe M, van Emden T, van Waardenburg V, Likic R, Richir M, van Agtmael MA, Moermond CTA, Tichelaar J. Environmentally sustainable prescribing: recommendations for EU pharmaceutical legislation. Lancet Planet Health. 2024;8(10):e715–6. 10.1016/S2542-5196(24)00230-4. 39303730

[39] PREMIER project. Prioritisation and Risk Evaluation of Medicines in the EnviRonment. 2020–2026. https://imi-premier.eu/. (retrieved 30.06.25).

[40] PSCI website. Driving responsible value chains in the Pharmaceutical Industry. https://pscinitiative.org/home. (retrieved 30.06.25).

[41] Puhlmann N, Vidaurre R, Kümmerer K. Designing greener active pharmaceutical ingredients: Insights from pharmaceutical industry into drug discovery and development. Eur J Pharm Sci. 2024;192:106614. 10.1016/j.ejps.2023.106614. 37858896

[42] Ramström H, Martini S, Borgendahl J, Ågerstrand M, Lärfars G, Ovesjö M-L. Pharmaceuticals and Environment: a web-based decision support for considering environmental aspects of medicines in use. Eur J Clin Pharmacol. 2020;76(8):1151–60. 10.1007/s00228-020-02885-1. 32388641 PMC7351842

[43] Realistic Medicine Team. Realistic Medicine. 2024. https://realisticmedicine.scot/. (retrieved 30.06.25).

[44] Riikonen S, Timonen J, Sikanen T. Environmental considerations along the life cycle of pharmaceuticals: Interview study on views regarding environmental challenges, concerns, strategies, and prospects within the pharmaceutical industry. Eur J Pharm Sci. 2024;196:106743. 10.1016/j.ejps.2024.106743. 38460610

[45] Robinson OC. Sampling in Interview-Based Qualitative Research: A Theoretical and Practical Guide. Qualitative Res Psychol. 2014;11(1):25–41. 10.1080/14780887.2013.801543.

[46] Rockström, J., Steffen, W., Noone, K., Persson, Å., Chapin III, F.S., Lambin, E., Lenton, T.M., Scheffer, M., Folke, C., Schellnhuber, H.J., Nykvist, B., Wit, C., Hughes, T.d., van der Leeuw, S., Rodhe, H., Sörlin, S., Snyder, P.K., Costanza, R., Svedin, U., Falkenmark, M., Karlberg, L., Corell, R.W., Fabry, V.J., Hansen, J., Walker, B., Liverman, D., Richardson, K., Crutzen, P., Foley, J. Planetary boundaries: exploring the safe operating space for humanity. Ecology and society. 2009;14 (2).

[47] Ryan EC, Dubrow R, Sherman JD. Medical, nursing, and physician assistant student knowledge and attitudes toward climate change, pollution, and resource conservation in health care. BMC Med Educ. 2020;20(1):200. 10.1186/s12909-020-02099-0. 32576175 PMC7310528

[48] Smith F. Health services research, methods in pharmacy. Qualitative interviews. Br J Clin Pharmacol. 1998;6 (2): 97–108. 10.1111/j.2042-7174.1998.tb00923.x.

[49] Steffen W, Richardson K, Rockström J, Cornell SE, Fetzer I, Bennett EM, Biggs R, Carpenter SR, Vries W, Wit CAd., Folke C, Gerten D, Heinke J, Mace GM, Persson LM, Ramanathan V, Reyers B, Sörlin S, editors. 2015. Planetary boundaries: Guiding human development on a changing planet. Science 347 (6223), 1259855. 10.1126/science.1259855. 25592418

[50] Sveriges Apoteksförening. Välvald. 2018. https://sverigesapoteksforening.se/valvald/. (retrieved 30.06.25).

[51] Sveriges Apoteksförening. Sector Report. 2023. https://sverigesapoteksforening.se/wp-content/uploads/2023/04/SVAP0011_Apoteksforeningen_Branschrapport_2023_ENG_Korr3.pdf. (retrieved 30.06.25).

[52] Thompson J. A guide to abductive thematic analysis. Qualitative Rep. 2022;27(5):1410–21. 10.46743/2160-3715/2022.5340.

[53] Thunberg S, Arnell L. Pioneering the use of technologies in qualitative research – A research review of the use of digital interviews. Int J Soc Res Methodol. 2022;25(6):757–68. 10.1080/13645579.2021.1935565.

[54] van der Zee J, Kroneman MW. Bismarck or Beveridge: a beauty contest between dinosaurs. BMC Health Serv Res. 2007;7(1):94. 10.1186/1472-6963-7-94. 17594476 PMC1934356

[55] van Wilder L, Boone L, Ragas A, Moermond C, Pieters L, Rechlin A, Vidaurre R, Smedt Dd, Dewulf J. A holistic framework for integrated sustainability assessment of pharmaceuticals. J Clean Prod. 2024;467:142978. 10.1016/j.jclepro.2024.142978.

[56] Vidaurre R, Bramke I, Puhlmann N, Owen SF, Angst D, Moermond C, Venhuis B, Lombardo A, Kümmerer K, Sikanen T, Ryan J, Häner A, Janer G, Roggo S, Perkins AN. Design of greener drugs: aligning parameters in pharmaceutical R&D and drivers for environmental impact. Drug Discovery Today. 2024;29(7):104022. 10.1016/j.drudis.2024.104022. 38750927

[57] von Soest C. Why Do We Speak to Experts? Reviving the Strength of the Expert Interview Method. Perspect Politics. 2023;21(1):277–87. 10.1017/S1537592722001116.

[58] Wilkinson, J.L., Boxall, A.B.A., Kolpin, D.W., Leung, K.M.Y., Lai, R.W.S., Galbán-Malagón, C., Adell, A.D., Mondon, J., Metian, M., Marchant, R.A., Bouzas-Monroy, A., Cuni-Sanchez, A., Coors, A., Carriquiriborde, P., Rojo, M., Gordon, C., Cara, M., Moermond, M., Luarte, T., Petrosyan, V., Perikhanyan, Y., Mahon, C.S., McGurk, C.J., Hofmann, T., Kormoker, T., Iniguez, V., Guzman-Otazo, J., Tavares, J.L., Gildasio De Figueiredo, F., Razzolini, M.T.P., Dougnon, V., Gbaguidi, G., Traoré, O., Blais, J.M., Kimpe, L.E., Wong, M., Wong, D., Ntchantcho, R., Pizarro, J., Ying, G.-G., Chen, C.-E., Páez, M., Martínez-Lara, J., Otamonga, J.-P., Poté, J., Ifo, S.A., Wilson, P., Echeverría-Sáenz, S., Udikovic-Kolic, N., Milakovic, M.e.a., Pharmaceutical pollution of the world’s rivers. Proceedings of the National Academy of Sciences of the United States of America. 2022;119 (8). 10.1073/pnas.2113947119. PMC8872717 35165193

