# Supplementary material for: Greener active pharmaceutical ingredients: perspectives of European healthcare professionals
Source: BMC Health Serv Res. 2026 Sep 4;26:1236. doi: 10.1186/s12913-026-15145-2 (PMC13551862; doi:10.1186/s12913-026-15145-2)
Supplement: Supplementary file 1 [file 12913_2026_15145_MOESM1_ESM.docx]

Supplementary Information

**Greener active pharmaceutical ingredients: Perspectives of European healthcare professionals**

Neele Puhlmann^a^, Yannick Heni^b^, Rodrigo Vidaurre^b^, Caroline T. A. Moermond^c,d^, Klaus Kümmerer ^a^, Tiina M. Sikanen^e^, Oliver Olsson^a^

^a^Institute of Sustainable Chemistry, Leuphana University of Lüneburg, Universitätsallee 1, 21335 Lüneburg, Germany

^b^Ecologic Institute, Pfalzburger Strasse 43/44, 10717 Berlin, Germany

^c^Centre for Safety of Substances and Products (VSP), National Institute for Public Health and the Environment (RIVM), P.O. Box 1, 3720 BA Bilthoven, The Netherlands

^d^Institute for Biosciences, Radboud University Nijmegen, Heyendaalseweg 135, 6225AJ, Nijmegen, Netherlands

^e^Faculty of Pharmacy, University of Helsinki, Viikinkaari 5E, 00790 Helsinki, Finland

e-mail corresponding authors: [oliver.olsson@leuphana.de](mailto:oliver.olsson@leuphana.de)

Content:

ANNEX I: Questionnaire for healthcare stakeholders

ANNEX II: Background summary for healthcare stakeholders

ANNEX III: Code scheme of the interview analysis

ANNEX I: Questionnaire for healthcare stakeholders

Interview questionnaire – Stakeholders’ perspectives on the use of greener Active Pharmaceutical Ingredients (greener APIs)

What is PREMIER?

PREMIER (Prioritisation and Risk Evaluation of Medicines In the EnviRonment; 2020-2026) is an Innovative Health Initiative (IHI) project, funded by the European Commission and members of the European Federation of Pharmaceutical Industries and Associations (EFPIA). The PREMIER consortium is made up of universities, research organisations, public bodies, non-profit groups and 13 pharmaceutical companies. The aim of PREMIER is to deliver a framework for assessing and characterising the environmental risks of Active Pharmaceutical Ingredients (APIs). Moreover, the goal of PREMIER Work Package 4.2 is to assess the feasibility of the design of greener APIs, and with this to contribute to one of the building blocks of sustainable healthcare systems.

Greener APIs are APIs that have less impact on the environment after the use phase (i.e., after excretion by patients and water treatment in sewage treatment plants). A fundamental principle behind any comparison of APIs’ environmental profiles is that patient well-being always comes first. We are exploring strategies to introduce environmental aspects into the drug discovery and development process, which may depend on factors external to the pharmaceutical industry, e.g. on the demand from the healthcare sector. The focus of our work is specifically limited to the API itself (thus excluding e.g. excipients and other ingredients of a pharmaceutical product) and its environmental impact after use (thus not including other aspects such as climate impacts, manufacturing emissions or resource use for production or packaging).

What is this interview about?

The interview aims to investigate perspectives of stakeholders of the healthcare sector on the topic of greener APIs, so as to understand the barriers and opportunities to incorporate this aspect in their work (e.g. favouring greener APIs in the healthcare sector). In particular, the one-hour interview focuses on:

i) the decision-making process of the stakeholder’s organisation in general,

ii) the current and future role of environmental considerations in this decision-making,

iii) factors that determine the feasibility of introducing environmental considerations in decision-making.

The results of this study will feed into a report evaluating the wider feasibility of greener APIs both from a design/manufacturing and a stakeholder uptake perspective (due in September 2024).

The accompanying short document Pharmaceuticals in the Environment – Understanding the Feasibility of Designing Greener APIs provides some background on the topic of pharmaceuticals in the environment, the understanding of greener APIs within PREMIER, and the research process in PREMIER Work Package 4.2.

Section 1: Setting the scene – Stakeholders’ decision-making processes related to the selection and use of pharmaceuticals

1. Please describe your organisation regarding:
a) its position in the life-cycle of pharmaceuticals (see figure in accompanying short document).
b) how its mandate is related to the selection/supply/use of pharmaceuticals.
2. Please describe your organisation’s decision-making relating to the selection/use of pharmaceuticals. (E.g.: How do you decide which pharmaceutical is selected/used?)

Section 2: Current and future role of environmental considerations

3. Do environmental aspects related to pharmaceuticals play a role in your decision-making regarding selection/use of pharmaceuticals?
   If “yes”:
a) Which ones, and which rationale was behind the uptake of these aspects?
b) How are these environmental aspects weighed in comparison to other criteria considered in the selection of pharmaceuticals?

Figure 1**^[1]^**: Removed due to copy rights. The figure showed that environmental sustainability is one pillar of overall sustainability (scope of question 3). ‘Ecotoxicological impact’ due to presence in the environment and effects (scope of questions 4 to 7) is one of the impact categories proposed to analyse sustainability of pharmaceuticals.

Now specifically on pharmaceuticals in the environment (after use/excretion) and effects:

4. What is your organisation’s knowledge regarding the topic “Pharmaceuticals in the environment and their effects” and potential measures?
5. Do post-use environmental impacts of pharmaceuticals play a role in your organisation’s decision-making?
a) If “yes”: Which impacts (most important) do you consider?
b) If “no”: Are considerations specific to such impacts expected to be included in your organisation’s future decision-making?
   - If “yes”: Which impacts may become relevant?

Q5a: (Only if your response to question Q5 was “yes” and your organisation is familiar with the aspects listed below.) Which parameters related to post-use environmental impacts may become relevant in your decision-making? Examples could be:

- Environmental Risk: PEC/PNEC
- Environmental exposure: emissions (e.g. concentrations in effluent at production site), environmental concentrations
- Hazardous properties: PBT, vPvB /PMT, vPvM
- Substance classification according to regulation, such as CLP and/or REACH
- Other parameters

a) Which drivers do you see behind your answer?
6. How important would you consider parameters on post-use environmental impacts when compared to other criteria, e.g., costs, dosing frequency, patient safety?

Example 1: Biologics are considered ‘greener’ alternatives (looking at the post-use impact) than traditional small molecules. However, costs and other considerations might be less favourable. What would your decision-making logic be in a case like this?

Example 2: Efforts in pharmaceutical R&D to achieve (a) once daily dosing, (b) minimizing safety issues associated to reactive metabolites, (c) avoiding UV-absorption (risk of phototoxicity), and (d) drug-drug interactions (altogether pushing for more metabolically stable substances), may have indirectly selected for drug substances that are also more stable in the environment. How important do you consider each of these aspects (a-d) and would you see room for compromising on any of them in the benefit of the environmental profile of a substance?

a) Would you follow the same decision-making logic when (a) a pharmaceutical has been associated to a confirmed environmental impact due to post-use releases and when (b) a pharmaceutical (despite a hazard classification) is concluded to represent no risk to the environment (e.g., environmental exposure remains lower than the level that could induce an impact)?
7. Do you see risks for undesired (unintended) consequences when selecting one API over another?
a) If “yes”: Please describe the kind of risks.
b) What would be required to avoid such risks?

Section 3: Factors that determine the feasibility of introducing environmental considerations in decision-making

8. Do your sustainability guidelines or policies refer to the use of pharmaceuticals? Is there any concept/approach in your organisation towards this, e.g. by selecting/prioritizing greener APIs?
9. What would be needed for your organisation to consider environmental aspects of APIs in future?
10. What kind of expertise is needed for assessing and weighing the various types of criteria, e.g. criteria for pharmaceutical’s application such as efficacy and safety, and criteria related to post-use environmental impacts?
a) Assuming expertise to perform a holistic assessment is available: Which healthcare actor(s) should perform the assessment (partly or fully) of post-use environmental impacts and/or the weighing of all different criteria?
   - Doctors
   - Pharmacists
   - Hospitals
   - Pharmaceutical Industry
   - Health system procurement agencies
   - Health insurance companies
   - Authorities responsible for marketing authorisations
   - Authorities involved in healthcare provision
   - Environmental authorities
   - Others

Last but not least

Could we contact you in case of follow-up questions?

If you are interested in further exchange, you can subscribe the [newsletter](https://imi-premier.eu/newsletter/), or the newsletter of the related Project [TransPharm](https://transforming-pharma.eu/subscribe). You can also contact us directly, e.g. to receive and discuss the report that includes the evaluation of this interview series.

ANNEX II: Background summary for healthcare stakeholders

Pharmaceuticals in the Environment – Understanding the Feasibility of the Use of Greener Active Pharmaceutical Ingredients

Authors: Leuphana University, Ecologic Institute, January 2023

This text aims to facilitate the exchange between stakeholders in the healthcare sector and the PREMIER consortium. It provides some background of the significance of the topic “Pharmaceuticals in the environment” with special focus on the environmental impact of the active pharmaceutical ingredient after use.

Introduction

Active Pharmaceutical Ingredients (APIs) and their metabolites are excreted into wastewater following patient use. Most APIs are only partly removed by conventional wastewater treatment and thus enter surface waters. Their presence in surface water and groundwater, i.e. drinking water sources, soils and sediments is linked to potential risks and increasingly driving public, stakeholder and regulatory concern. Ecotoxicological effects of APIs have been shown to occur at environmentally relevant concentrations in laboratory studies. Also, effects of sewage treatment effluent (which includes pharmaceutical residues and other micropollutants) on populations of organisms in surface water have been shown.

Because of these concerns, several EU strategies and resolutions as well as global initiatives (e.g. by [UNEP](https://saicmknowledge.org/about/about), [OECD](https://www.oecd.org/publications/pharmaceutical-residues-in-freshwater-c936f42d-en.htm)) have focused on addressing pharmaceuticals in the environment. For example, the EU will monitor an increasing number of APIs under its Water Framework Directive and in 2020 it adopted the ‘[Pharmaceutical Strategy for Europe](https://ec.europa.eu/commission/presscorner/detail/en/ip_20_2173)’, where one of the focus points is the environmental impact of pharmaceuticals. Moreover, the [draft proposal](https://health.ec.europa.eu/medicinal-products/pharmaceutical-strategy-europe/reform-eu-pharmaceutical-legislation_en) of a new Directive and a new Regulation (published by the European Commission on 26 April 2023), which will revise and replace the existing general pharmaceutical legislation, includes changes under which mitigation of environmental impact would be a more stringent part of the approval decision of medicinal products. In addition, regulatory initiatives addressing pollution in general can also have relevance for the pharmaceutical sector, such as the zero pollution ambition announced under the [European Green Deal](https://commission.europa.eu/strategy-and-policy/priorities-2019-2024/european-green-deal_en), which aims to protect citizens and ecosystems from pollution. For instance, and supporting this ambition, a proposal to ban per- and polyfluoroalkyl substances ([PFAS](https://echa.europa.eu/-/echa-publishes-pfas-restriction-proposal)) was developed under the EU chemicals regulation REACH, which could become relevant in future for the pharmaceutical sector.

PREMIER (Prioritisation and Risk Evaluation of Medicines in the EnviRonment; 2020-2026) is an Innovative Health Initiative (IHI; formerly IMI) project, funded by the European Commission and pharmaceutical companies that are members of the European Federation of Pharmaceutical Industries and Associations (EFPIA). PREMIER consists of universities, research organisations, public bodies, non-profit groups, small-to-medium-enterprises, and 13 pharmaceutical companies; Radboud University and AstraZeneca are the academic and industry leaders of the consortium. For more information on the project, please refer to <https://imi-premier.eu/>.

From Design to Use of greener APIs

PREMIER explores the feasibility of various approaches for reducing environmental risks of pharmaceutical residues. One way to reduce the presence of APIs in the environment could be the design of greener APIs. Possible approaches could include innovative therapies, e.g. precision or personalised medicines. Reduced environmental impact could also be achieved by including environmental criteria in the design and selection of API candidates during the drug discovery and development process. Importantly, the introduction of greener criteria needs to align with existing drug discovery principles and be balanced against the patients’ benefit/risk. In other words, a greener API needs to be a clinically efficacious drug.

The current understanding of the PREMIER consortium is that greener APIs “are inherently less harmful for the environment after use due to reduced potential for environmental exposure (reduced persistence or potential to reach some environmental compartments) and/or due to reduced environmental effects” (see below, Supplementary Figure 1). Using the comparative ‘greener’ indicates that this is not an ‘absolute property’ but a way to designate a significant improvement in the environmental profile when compared to other analogues or relevant APIs. How ‘green’ an API should be to avoid unacceptable environmental risks will largely depend on the use pattern and resulting emissions” (see below > further reading [1]). The focus of the PREMIER project is on the risks posed by pharmaceuticals entering the environment. Environmental and climate impacts resulting from manufacturing are out of scope of PREMIER and thus also out of scope of this interview series with stakeholders of the healthcare sector.

One goal of PREMIER is to assess the feasibility of the design of greener APIs. Within this work package, we gained insights from drug design experts and analysed the feasibility of strategies to incorporate environmental parameters into the drug discovery and development process. This included the identification of opportunities and barriers, e.g. challenges related to the current state of knowledge and the availability of suitable screening tests that can contribute to the assessment of environmental parameters. Incentives are needed to overcome barriers for the design of greener APIs (see below > further reading [1,2]). As a next step, we want to investigate the demand, degree of readiness and potential use of greener APIs by relevant stakeholders to complement the wider picture across the healthcare sector. To achieve meaningful input from across all of the wider healthcare sector, representatives from all relevant stakeholder groups should be identified, reached out to and given opportunity to participate in these interviews. This is crucial for the comprehensiveness of the gathered inputs and to conduct a realistic analysis of the feasibility of designing and developing greener APIs. For this reason, we want to interview relevant stakeholders in the healthcare sector, including general practitioners, pharmacists, health system procurement agencies, health insurers, public health authorities, authorities involved in healthcare provision, and sustainability reporting teams from pharmaceutical companies. We also plan to interview representatives of consumers and environmental NGOs.

Interviews with stakeholders in healthcare

In February 2024, [Leuphana University of Lüneburg](https://www.leuphana.de/en/home.html) and [Ecologic Institute](https://www.ecologic.eu/) will start performing structured interviews with stakeholders in the healthcare sector. They are aimed to investigate perspectives of interviewed stakeholders on:

i) the decision-making process of the stakeholder in general

ii) the current and future role of environmental considerations in this decision-making

iii) factors that determine the feasibility of introducing environmental considerations in decision-making

These interviews (ca. 1 hour per stakeholder/interview) will be performed with representatives of the stakeholder groups listed previously. Each interview will be held separately with representatives of the same stakeholder group. We welcome the presence of more than one representative from each stakeholder group, as this can lead to more in-depth responses. The questionnaire will be provided at least one week before the interview.

Research outputs will be summarised in a project report discussing the feasibility of the design of greener APIs (due date is September 2024). If needed, non-disclosure agreements can be agreed upon and signed.

Supplementary Figure 1: GREENER concept for discovery and development of APIs with lower impact on the environment after use by patients (Moermond et al., 2022).

For further reading

1. Greener APIs
   - Article, [GREENER Pharmaceuticals for More Sustainable Healthcare](https://doi.org/10.1021/acs.estlett.2c00446)
   - Summary report, [describing fundamental drug design principles and their environmental significance](https://imi-premier.eu/wp-content/uploads/2023/03/D4.6_WP-4-Greener-Drug-Design-Report_FINAL-130323.pdf) (short scientific article based on this report was written; soon under review)
2. Further insights from PREMIER project
   - •	Article, [Designing greener active pharmaceutical ingredients: Insights from pharmaceutical industry into drug discovery and development](https://doi.org/10.1016/j.ejps.2023.106614)
   - Report, [summarising drug discovery and development process and key attributes of tools/models (D4.2.1)](https://cordis.europa.eu/project/id/875508/results)
3. More Background
   - [Report on environmental risks posed by medicinal products](https://ec.europa.eu/health/sites/health/files/files/environment/study_environment.pdf), by BIO IS for the EU Commission
   - [Key publication](https://www.researchgate.net/publication/333678147_Pharmacology_beyond_the_patient_-The_environmental_risks_of_human_drugs) on ecological risks of pharmaceuticals
   - Publication on [design of pharmaceuticals for environmental biodegradability](https://doi.org/10.1016/j.scp.2019.100136)

ANNEX III: Code scheme of the interview analysis

Supplementary Table 1: Code scheme of the interview analysis

| Name of Code | | | | Information | Frequency  absolute│ in % | |
| --- | --- | --- | --- | --- | --- | --- |
| Total | | | |  | 698 | 100 |
|  | Privacy Policy | | | Agreement with the privacy policy that was sent beforehand | 18 | 3 |
|  | Project Intro | | | We introduced the project and ourselves | 16 | 2 |
|  | Section 1 | | | Q1: Setting the scene – Stakeholders’ decision-making processes related to the selection and use of pharmaceuticals |  |  |
|  |  | SH’s life cycle position | | Q1a: Please introduce yourself and describe your organisation regarding: its position in the life-cycle of pharmaceuticals (see figure in accompanying short document). | 34 | 5 |
|  |  | SH’s mandate | | Q1b: Please introduce yourself and describe your organisation regarding: how its mandate is related to the selection/supply/use of pharmaceuticals. | 30 | 4 |
|  |  | Decision-Making Process | | Q2: Description of organisation’s decision-making relating to the selection/use of pharmaceuticals. | 46 | 7 |
|  | Section 2 | | | Current and future role of environmental considerations |  |  |
|  |  | Role of Envi Aspects | | Q3: Do environmental aspects related to pharmaceuticals play a role in your decision-making regarding selection/use of pharmaceuticals? | 48 | 7 |
|  |  |  | Aspects and Rationale  for Uptake | Q3a: Which Envi Aspects play a role currently, and which rationale was behind the uptake of these aspects? | 56 | 8 |
|  |  |  | Weighing | Q3b: How are current environmental aspects weighed in comparison to other criteria considered in the selection of pharmaceuticals? | 24 | 3 |
|  |  | Knowledge on PiE | | Q4: Organisation’s knowledge regarding the topic “Pharmaceuticals in the environment and their effects” and potential measures? | 33 | 5 |
|  |  | Role of Post-Use | | Q5: Do post-use environmental impacts of pharmaceuticals play a role in your organisation’s decision-making? | 28 | 4 |
|  |  |  | Post-use impacts | Q5a: Which post-use environmental impacts of pharmaceuticals (most important) do you consider? | 13 | 2 |
|  |  |  | Expectation | Q5b: Are considerations specific to such impacts expected to be included in your organisation’s future decision-making? If “yes”: Which impacts may become relevant? | 31 | 4 |
|  |  | Parameters | | Q5A: Which parameters related to post-use environmental impacts may become relevant in your decision-making? Examples could be:   - Environmental Risk: PEC/PNEC - Environmental exposure: emissions (e.g. concentrations in effluent at production site), environmental concentrations - Hazardous properties: PBT, vPvB /PMT, vPvM - Substance classification according to regulation, such as CLP and/or REACH - Other parameters   Q5A.a: Which drivers do you see behind your answer | 48 | 7 |
|  |  | Weighing - Envi, Costs,  Patient aspects | | Q6: How important would you consider parameters on post-use environmental impacts when compared to other criteria, e.g., costs, dosing frequency, patient safety? | 52 | 7 |
|  |  |  | Risk vs. Hazard | Q6a: Would you follow the same decision-making logic when (a) a pharmaceutical has been associated to a confirmed environmental impact due to post-use releases and when (b) a pharmaceutical (despite a hazard classification) is concluded to represent no risk to the environment (e.g., environmental exposure remains lower than the level that could induce an impact)? | 18 | 3 |
|  |  | Unintended Consequences | | Q7a: Do you see risks for undesired (unintended) consequences when selecting one API over another?  Please describe the kind of risks. | 30 | 4 |
|  |  |  | Avoiding Risks | Q7b: What would be required to avoid such risks? | 21 | 3 |
|  | Section 3 | | | Factors that determine the feasibility of introducing environmental considerations in decision-making |  |  |
|  |  | Sustainability Guideline | | Q8: Do your sustainability guidelines or policies refer to the use of pharmaceuticals? Is there any concept/approach in your organisation towards this, e.g. by selecting/prioritizing greener APIs? | 23 | 3 |
|  |  | Future Needs | | Q9: What would be needed for your organisation to consider environmental aspects of APIs in future? | 41 | 6 |
|  |  | Expertise | | Q10: What kind of expertise is needed for assessing and weighing the various types of criteria, e.g. criteria for pharmaceutical’s application such as efficacy and safety, and criteria related to post-use environmental impacts? | 21 | 3 |
|  |  |  | Key-Player | Q10a: Assuming expertise to perform a holistic assessment is available: Which healthcare actor(s) should perform the assessment (partly or fully) of post-use environmental impacts and/or the weighing of all different criteria?   - Doctors - Pharmacists  Hospitals - Pharmaceutical Industry - Health system procurement agencies - Health insurance companies - Authorities responsible for marketing authorisations - Authorities involved in healthcare provision - Environmental authorities - Others | 32 | 5 |
|  | Follow-Up Questions | | | Q11: Can be contacted if there are follow-up questions. | 16 | 2 |
|  | Out of Scope | | | e.g., on production | 8 | 1 |
|  | Not relevant | | | Formalities, Technical Issues, without content | 11 | 2 |

1. Taken from van Wilder et al. (2024). A holistic framework for integrated sustainability assessment of pharmaceuticals. Journal of Cleaner Production 467, 142978. doi: 10.1016/j.jclepro.2024.142978.
